# Subchronic Exposure to Hexavalent Chromium in Drinking-Water-Induced Sex-Dependent Essential Metal Dyshomeostasis in the Liver, Blood, and Brain of Guinea Pigs

**DOI:** 10.3390/livers6050090

**Published:** 2026-09-04

**Authors:** Morgan E. Delnicki, Samuel T. Vielee, Gabrielle C. Griffin, Idoia Meaza, Olusegun Ogunsuyi, Serigne Fallou Gueye, Aggie Brownell, Nicolli Butzke-Souza, Jack Easley, Haiyan Lu, Sandra S. Diven, J. Calvin Kouokam, John P. Wise, William J. Buchanan, Elizabeth Evans, Natalie Besaw, Hamza Jamal, John Pierce Wise, Jamie L. Wise

**Affiliations:** 1 Wise Laboratory of Liver and Environmental Health, Department of Pharmacology and Toxicology, University of Louisville, Louisville, KY 40202, USA; 2 Pediatric Research Institute, University of Louisville, Louisville, KY 40202, USA; 3 Mote Marine Laboratory, Sarasota, FL 34236, USA; 4 Wise Laboratory of Environmental and Genetic Toxicology, Department of Pharmacology and Toxicology, University of Louisville, Louisville, KY 40202, USA; 5 NeuroWise Laboratory, Department of Pharmacology and Experimental Therapeutics, The University of Toledo, Toledo, OH 43606, USA

**Keywords:** hexavalent chromium, essential metal dyshomeostasis, guinea pig, drinking water exposure, liver, blood, brain

## Abstract

**Background/Objectives:**

Hexavalent chromium [Cr(VI)] is a toxic metal that enters the environment due to natural and anthropogenic processes. The liver is a major target organ of Cr(VI) exposure through drinking water, resulting in an increased risk of exposed individuals developing chronic liver diseases and cancers. Cr(VI) exposure is also associated with neurological dysfunction. Essential metal dyshomeostasis is a well-characterized biomarker of liver and neurological diseases, but the role Cr(VI) plays in altering essential metal levels in both organs is poorly understood.

**Methods:**

Male and female Hartley guinea pigs were exposed to 0 or 5 mg Cr(VI)/L in drinking water for 90 days. Chromium accumulation and essential metal levels in the liver, blood and brain were analyzed using inductively coupled plasma mass spectrometry.

**Results:**

Chromium significantly accumulated in a sex-dependent manner in the livers and blood of guinea pigs given 5 mg Cr(VI)/L in their drinking water, with sex- and region-specific chromium deposition observed in the brain. The livers and blood of female guinea pigs were more vulnerable to Cr(VI) exposure, as evident in the significant chromium accumulation and dyshomeostasis of several essential metals, including hepatic magnesium, zinc and potassium. Conversely, male guinea pigs were more vulnerable to Cr(VI)-induced essential metal dyshomeostasis in the brain, with significant changes in molybdenum and cobalt in nearly half of the 11 brain regions assessed.

**Conclusions:**

This study begins the process of establishing a novel model to study Cr(VI)-induced hepatotoxicity, neurotoxicity and organ crosstalk. Overall, Cr(VI) induced sex-dependent essential metal dyshomeostasis in the liver and blood, along with sex- and region-specific essential metal dyshomeostasis in the brain. These results suggest essential metal dyshomeostasis may play an important role in Cr(VI)-induced liver and neurodegenerative diseases, and these effects should guide future mechanistic investigations.

## 1. Introduction

Hexavalent chromium [Cr(VI)] is an environmental toxicant of major public health concern. Cr(VI) is listed as a “Top 20 Hazardous Substance” on the Agency for Toxic Substances and Disease Registry’s “Substance Priority List” and was identified as the number one chemical hazard in U.S. public drinking water [1,2]. Cr(VI) can enter the environment via geogenic sources (e.g., ultramafic igneous bedrock) found in U.S. southwestern states (California, New Mexico, and Arizona) and the Piedmont region along the East Coast (Alabama to New Jersey) [2–5], as well as through anthropogenic activities [2–7]. Drinking water is at an elevated risk of Cr(VI) contamination due to industrial run-off settling in downstream drinking-water basins and our aging water infrastructure (corroding cast iron pipes) [8,9]. In fact, water supplies for over 200 million Americans are contaminated with Cr(VI), according to federal data [10]. Upon drinking-water exposure, Cr(VI) notably targets and accumulates in the liver [6]. Epidemiological data indicate there is a correlation between drinking-water Cr(VI) exposure and an increased incidence of liver disease and primary liver cancers [11].

Exposure to toxic metals, such as Cr(VI), contributes to dyshomeostasis of essential metal levels. These essential metals are involved in a variety of biological processes vital for maintaining optimal function of an organism [12,13]. For example, zinc is an antioxidant metal that is a cofactor in several enzymes responsible for reducing oxidative stress, a known contributor to the development of chronic diseases [14]. Electrolytes such as sodium and calcium are essential metals that facilitate pivotal cellular processes, including nerve impulse conduction and muscle contraction, respectively [15,16]. Few studies have assessed the impact of Cr(VI) on essential metal dyshomeostasis [17–19]. One study found that 90 days of exposure to Cr(VI) in drinking water induced age-, sex- and region-dependent essential metal dyshomeostasis in the brains of Sprague–Dawley rats [17]. Another study found that oropharyngeal exposure to particulate Cr(VI) induced essential metal dyshomeostasis in the lung, liver, blood and brain in an exposure- and sex-dependent manner in guinea pigs [18,19]. However, there are currently no studies assessing the impact Cr(VI)-contaminated drinking water has on essential metal dyshomeostasis in the liver, blood, and brain of guinea pigs.

The majority of the published in vivo Cr(VI) drinking-water studies were performed using rats or mice [6]. However, there are key physiological differences between rats, mice, and humans that suggest these studies may have limited translation to human outcomes [20,21]. This translational gap presents the need for a robust Cr(VI) drinking-water model where findings can be better translated to human populations, such as guinea pigs. Hartley guinea pigs (*Cavia porcellus*) have a rich history in research, are often utilized as models for various respiratory diseases and osteoarthritis, and possess many unique characteristics that make them better models for the purpose of translating findings into human populations [22–24].

The physiology of a guinea pig liver differs greatly from those of rats and mice and is much more similar to a human liver. The key distinction is that, like humans, guinea pigs are unable to endogenously synthesize ascorbic acid (vitamin C) and must obtain it through their diet [25]. This is an important consideration for Cr(VI) studies, as vitamin C is an antioxidant that can extracellularly reduce toxic Cr(VI) to non-toxic Cr(III) [26,27]. Endogenously produced vitamin C in rats and mice could limit Cr(VI) distribution and tissue deposition, making the animals more resistant to and protected against Cr(VI) toxicity, compared to humans. Recent studies have also identified the liver as the major organ involved in the clearance of neurodegenerative pathologies, particularly plasma amyloid beta, from circulation [28,29]. One mechanism in which Cr(VI) is believed to induce hepatotoxicity is through the generation of reactive oxidative species that induce inflammation in the liver [30–32]. Hepatic inflammation greatly reduces the ability of the liver to clear plasma amyloid beta from circulation, allowing for re-entry into the brain and subsequent amyloid beta plaque deposition that is involved in the pathogenesis of neurodegenerative diseases [33–35]. However, there is a major knowledge gap in understanding the crosstalk that occurs between the liver and brain, and the potential role Cr(VI) plays in disrupting this crosstalk. The lack of data on liver–brain crosstalk is especially concerning given the epidemiological data showing major correlations in individuals that have both liver disease and neurodegenerative diseases [36–39]. One way to investigate crosstalk between these organs is to quantify essential metal levels, as essential metal dyshomeostasis has recently been identified as a potential biomarker for both liver disease and neurodegeneration [40–42]. Therefore, we developed a novel drinking-water model for Cr(VI) exposure using Hartley guinea pigs to investigate the impact of Cr(VI) on essential metal levels in the liver, blood and brain as the first step in characterizing the impact of Cr(VI) on liver–brain crosstalk.

## 2. Materials and Methods

### 2.1. Animals

This animal study was approved by the Institutional Animal Care and Use Committee (IACUC) (#24423) of the University of Louisville and complied with all IACUC animal use, protocol and procedure guidelines. In sum, 20 male and 20 female retired breeder Hartley guinea pigs, aged 10.5–11.5 months, were purchased from Charles River (Wilmington, MA, USA). The guinea pigs were housed in a temperature- and light-controlled room with a 12 h light/dark cycle, in facilities accredited by the Association for Assessment and Accreditation of Laboratory Animal Care.

Upon arrival, guinea pigs were given two weeks to acclimate to the facility. After the first week, guinea pigs were briefly sedated with vaporized isoflurane (Henry Shien; Indianapolis, IN, USA), and a microchip was inserted subcutaneously into each guinea pig for identification purposes. Guinea pigs were provided food and water *ad libitum*. The guinea pigs were fed a certified diet (Teklad Global Guinea Pig Diet 2040C, Inotiv; West Lafayette, IN, USA) throughout the study. Body mass was measured weekly, and water volume was measured twice daily in the mornings and afternoons. During the acclimation period, three guinea pigs were lost due to attrition, including one male from the control group and two females from the control group, bringing the total number of guinea pigs to 37, divided as evenly as possible into the following experimental groups: control male guinea pigs (*n* = 9); control female guinea pigs (*n* = 8); Cr(VI)-exposed male guinea pigs (*n* = 10); and Cr(VI)-exposed female guinea pigs (*n* = 10). No exclusion criteria were set.

### 2.2. Cr(VI) Exposure

After acclimation, guinea pigs were separated into sex- and body mass-matched control and Cr(VI)-exposed groups for a total of four experimental groups. Guinea pigs received tap water or 5 mg Cr(VI)/L in tap water in the form of sodium chromate (Na_2_CrO_4,_ CAS #7775-11-3, 98% purity, Sigma Aldrich; St. Louis, MO, USA) in drinking water, *ad libitum*, for 90 days. Cr(VI) exposures were prepared and diluted as needed from a 5000 mg Cr(VI)/L stock solution to a final exposure concentration of 5 mg Cr(VI)/L.

A quantity of 5 mg Cr(VI)/L was chosen as the exposure concentration, based on levels of Cr(VI) measured in private well water reported out of Midland, TX. In 2009, the Texas Commission on Environmental Quality (TCEQ) was contacted by residents due to concerns over yellow tap water. This led to an investigation that discovered that 28 out of 50 private wells tested had levels of total Cr that exceeded the Environmental Protection Agency’s current maximum contaminant level of 0.1 mg/L, with the highest reported level being 5.41 mg Cr(VI)/L [43]. Furthermore, many previous Cr(VI) drinking-water studies using rodent models utilized exposure concentrations of Cr(VI) far exceeding levels of what a human would likely be exposed to, with concentrations of up to 516 mg Cr(VI)/L, and in some instances 1000 mg Cr(VI)/L was used [44–48]. The exposure ran for 90 days to model subchronic exposure to Cr(VI) through drinking water. The full experimental design and exposure paradigm can be seen in Figure 1.

### 2.3. Euthanasia

Guinea pigs were anesthetized and euthanized following adaptations of previously published methods [17,18]. Briefly, guinea pigs were anesthetized via intraperitoneal injection with 100 mg/mL xylazine and 100 mg/mL ketamine (combined at a 1:12 ratio of ketamine: xylazine) at a dose of 240 mg/kg body mass at the end of 90 days. Guinea pigs were then euthanized via exsanguination, using cardiac puncture and perfusion through the right ventricle with 450–560 mL cold 1X Dulbecco’s phosphate-buffered saline (DPBS), without calcium or magnesium (Corning, New York, NY, USA) [18].

### 2.4. Tissue Sample Collection

Whole blood was collected directly from the right ventricle into trace element-free K_2_ EDTA BD Vacutainer^®^ tubes (BD, Franklin Lakes, NJ, USA) prior to perfusion; these were stored at −80 °C until metallomic analyses, as described in previously reported methods [18]. Following exsanguination, guinea pigs were decapitated. The brains were extracted, rinsed in 1X DPBS, and blotted to remove excess liquid. The brains were then bisected using a guinea pig brain matrix (Visikol, Hampton, NJ, USA). One brain hemisphere from every other animal (5 control males, 5 exposed males, 4 control females, and 5 exposed females) was micro-dissected. Specially, the amygdala, brain stem, cerebellum, entorhinal cortex, frontal cortex, dorsal hippocampus, ventral hippocampus, hypothalamus, olfactory bulb, pituitary gland, and striatum were isolated referencing a rat brain atlas, as there is no published guinea pig brain atlas. The cerebellum, olfactory bulb, and hypothalamus were isolated before further dissection. Next, the brain stem was pulled to the rostral position to expose the white matter where the brain stem meets the midbrain. The white matter was cut at this connection to isolate the brain stem. Tapered spatulas were placed under the hippocampus and pulled caudally to isolate it from the cortex; the hippocampus was then cut sagittally to separate the dorsal and ventral hippocampus. The striatum was accessed by retracting the white matter at the rostral position to access the cortex. Similarly, the white matter at the medial ventral portion of the brain was retracted to obtain the amygdala. The cortex was cut coronally to isolate the frontal cortex, then cut at an angle across the caudal ventral portion to isolate the entorhinal cortex. Microdissections were performed under the supervision of an experienced neurotoxicologist. All tools were decontaminated using 70% ethanol. Isolated brain regions were stored in 15 mL trace element-free tubes for metallomic analyses. Brain regions were stored at −80 °C until analysis. Livers were extracted, rinsed in 1× DPBS, and blotted to remove excess liquid according to previously published methods [18]. Liver samples from the quadrate lobe were snap-frozen in liquid nitrogen and stored at −80 °C until metallomic analyses.

### 2.5. Inductively Coupled Plasma-Mass Spectrometry (ICP-MS) Analysis

Liver and blood samples were sent to collaborators at the Integrative Molecular Analysis Core (IMAC) at the University of New Mexico for ICP-MS analysis to quantify chromium, iron, copper, magnesium, cobalt, manganese, zinc, molybdenum, sodium, calcium, and potassium. Liver samples were digested in 0.3 mL of 70% nitric acid at 100 °C for 2 h. After the samples were digested, 5 mL of ultrapure water was added to dilute the samples. Separately, 0.5 mL of whole blood was digested in 0.5 mL 70% nitric acid at 100 °C for 2 h. Digested blood samples were then diluted to 10 mL using ultrapure water. Samples were analyzed using an Agilent 7900 ICP-MS. Calibration curves were established for each metal of interest based on a blank sample and an Environmental Calibration Standard (Agilent, Santa Clara, CA, USA). Samples were run alongside reference standards for trace elements in water (NIST1643F, Millipore Sigma, Burlington, MA, USA). Initial calibration blank and initial calibration verification standards were run at the beginning of the analysis. Quality control samples were integrated into data acquisition and analysis after every 10 samples (Table S1). An internal standard was included in all sample runs to account for any matrix effects. There were no reported values below the method’s detection limit. Liver metal values are reported as nanogram of metal per gram of wet tissue (ng/g), and blood metal values are reported as nanogram of metal per milliliter (ng/mL) of whole blood.

Brain regions were analyzed by the Metallomics Core Facility at the University of Louisville for the quantification of chromium, iron, copper, magnesium, cobalt, manganese, zinc, molybdenum, sodium, calcium, and potassium. Brain regions were digested in 70% nitric acid for 3 h at 80 °C. Tissues were then cooled to room temperature before adding 30% hydrogen peroxide and digesting for an additional hour at 80 °C. Digested brain samples were diluted to 5% nitric acid in Millipore water and filtered through Acrodisc 32 mm, 0.45 um Supor^®^ filters (Pall Corporation, Port Washington, NY, USA) into trace element-free 15 mL centrifuge tubes (VWR Avantor, Radnor, PA, USA). Digested and filtered samples were stored at −20 °C until metals analyses. Samples were analyzed using an Agilent 7800 ICP-MS. Calibration curves were established for each metal of interest based on a blank sample and an Environmental Calibration Standard (Inorganic Ventures, Christianburg, VA, USA). Quality control samples were integrated into data acquisition and analysis after every 20 samples (Table S2). An internal standard was included in all sample runs to account for any matrix effects. There were no reported values below the method’s detection limit. Brain metal values are reported as nanogram of metal per gram of wet tissue (ng/g).

### 2.6. Statistical Analysis

The distribution of the data was analyzed using a Shapiro–Wilks normality test. For normally distributed data, differences between groups were assessed using a two-tailed Student’s *t*-test with Welch’s correction (parametric). For non-normally distributed data, comparisons between groups were made using a Mann–Whitney U test (non-parametric). Significance was reported for *p*-values < 0.1 to account for the greater genetic variability seen among individual Hartley guinea pigs, and to show relationships that have a 90% or greater chance of being significant. All graphs and statistical analyses were performed using GraphPad Prism (ver. 10.3.1). Data are reported as mean ± standard error of the mean.

## 3. Results

### 3.1. Subchronic Exposure to Cr(VI) Altered the Body and Organ Mass of Guinea Pigs

Male guinea pigs exposed to Cr(VI) gained the most mass over time and maintained a higher body mass than their sex-matched controls for the duration of the study (Figure 2A). Female guinea pigs exposed to Cr(VI) also gained more mass compared to their sex-matched controls, but this difference was not obvious until week 4 (Figure 2B). Specifically, control male guinea pigs had a 10.8% increase in body mass by the end of the study, compared to exposed male guinea pigs, which had a 12.7% increase in body mass. Comparatively, control female guinea pigs had a 12.2% increase in body mass at the end of the study, while exposed female guinea pigs had a 15.0% increase in body mass. There is currently no explanation as to why Cr(VI)-exposed animals had higher body masses compared to their control counterparts, and this finding warrants further investigation. To further investigate the impact Cr(VI) had on whole body measurements, organ masses were normalized to the tibia length. Comparing organ mass to tibia length is a method commonly used to assess toxicant-induced changes, as the organ mass should remain proportional to the tibia length for each guinea pig. No significant changes in the liver-to-tibia ratios or whole-brain-to-tibia ratios were observed when comparing control guinea pigs to Cr(VI)-exposed guinea pigs (Figure 3A,B). No notable sex differences were seen in the liver-to-tibia ratios or brain-to-tibia ratios (Figure 3C,D).

### 3.2. Exposed Male and Female Guinea Pigs Consumed Equivalent Amounts of Cr(VI) from Their Drinking Water

Water consumption was monitored for the entire 90 days of the study. The total amount of Cr(VI) each guinea pig consumed in milligrams was estimated by multiplying the average total water consumption in liters by five to account for the final concentration of 5 mg Cr(VI)/L. All guinea pigs were housed in pairs upon arrival; however, single housing became necessary when fighting resulted in injury. Notably, all male control animals were housed separately, while half of the Cr(VI)-exposed males were housed in pairs. All females, independent of exposure, were housed in pairs throughout the study. This information is important when assessing water consumption. Control male guinea pigs consumed the most water, compared to all other experimental groups, over the course of the study (Figure 4A). Guinea pigs often manipulate their water bottles and spill water into their cages [49]; therefore, we speculate that because control male guinea pigs were housed alone, they manipulated their water bottles more, possibly out of boredom, causing them to spill more and appear as though they drank more water. However, exposed male and female guinea pigs consumed, on average, an equivalent amount of water during the study (Figure 4A). Exposed male and female guinea pigs also consumed similar amounts of Cr(VI) (Figure 4B).

### 3.3. Total Chromium Tissue Levels Increased in a Sex-Dependent Manner in Exposed Guinea Pigs

Total chromium levels were measured in liver, whole blood, and brain tissues, using ICP-MS. Chromium significantly accumulated in the livers of guinea pigs given 5 mg Cr(VI)/L in drinking water for 90 days (Figure 5A). Specifically, exposed guinea pigs accumulated an average of 31.80 ng/g chromium in their liver, while 4.70 ng/g of hepatic chromium was detected in control guinea pigs. When considering sex, exposed female guinea pigs accumulated more chromium in their liver compared to males (Figure 5B). Specifically, an average of 43.20 ng/g of chromium (a 9.5-fold increase compared to sex-matched controls) accumulated in the livers of exposed females, compared to exposed male livers, which contained an average of 20.39 ng/g of chromium (a 4.2-fold increase compared to sex-matched controls).

To gain a better understanding of the systemic distribution that occurs with drinking-water Cr(VI) exposure, whole blood samples were analyzed for chromium. Similar to the findings for the liver, chromium levels significantly increased in the blood of guinea pigs given 5 mg Cr(VI)/L in their drinking water for 90 days (Figure 5C). On average, exposed guinea pigs had 13.33 ng/mL of chromium in whole blood, compared to controls, which had an average of 3.80 ng/mL of chromium in the whole blood. A significant sex difference was noted when comparing males and females exposed to 5 mg Cr(VI)/L in their drinking water (Figure 5D). Specifically, exposed females had an average of 18.30 ng/mL of chromium in their blood (a 6.3-fold increase compared to female controls), while whole blood samples from exposed males only contained an average 8.36 ng/mL of chromium (a 1.8-fold increase compared to male controls).

Chromium levels were measured across 11 brain regions (i.e., amygdala, brain stem, cerebellum, entorhinal cortex, frontal cortex, dorsal hippocampus, ventral hippocampus, hypothalamus, olfactory bulb, pituitary gland and striatum). However, chromium only accumulated in the dorsal hippocampus and the pituitary gland (Figure 5E–H). Specifically, exposed guinea pigs had more than double the amount of chromium in their dorsal hippocampi compared to control guinea pigs, with an average of 38.19 ng/g chromium in the exposed guinea pigs compared to 15.48 ng/g chromium in control guinea pigs (Figure 5E). When considering sex, exposed females accumulated 5.4 times more chromium in their dorsal hippocampi, compared to sex-matched controls (Figure 5F). Conversely, chromium did not significantly accumulate in the dorsal hippocampus of exposed male guinea pigs. Chromium also accumulated in the pituitary glands of exposed guinea pigs (Figure 5G). Comparing across exposure groups, the average chromium content in the pituitary glands of control guinea pigs was 70.07 ng/g, while exposed guinea pigs had an average of 96.33 ng/g chromium in the pituitary gland. A sex difference was once again observed, with females having an average of 102.30 ng/g chromium in their pituitary glands (a 2-fold increase compared to sex matched controls), while no significant increase was seen in chromium levels in the pituitary glands of exposed male guinea pigs (Figure 5H). Taken together, these data suggest that chromium accumulation in the dorsal hippocampus and pituitary gland is largely driven by observations in the female guinea pigs. No significant chromium accumulation was observed in any of the other isolated brain regions (Figure S1).

### 3.4. Subchronic Exposure to Cr(VI)-Induced Essential Metal Dyshomeostasis in the Guinea Pig Liver, Blood and Brain

Levels of essential metals in the liver, blood and brain (including sodium, magnesium, potassium, calcium, manganese, iron, cobalt, copper, zinc and molybdenum) were quantified using ICP-MS. An essential metal was considered to be in dyshomeostasis when the mean metal level in exposed guinea pigs was statistically significantly different from the mean metal level of the sex-matched control. Changes in essential metal levels were then represented as fold-change relative to sex-matched controls for the liver, blood and brain.

In the liver, Cr(VI) exposure significantly decreased magnesium, potassium, and zinc levels (Figure 6A,C,E). While these decreases were observed in both sexes, they were only significantly decreased in the female guinea pigs (Figures 6B,D,F and 7). For example, females exposed to Cr(VI) in their drinking water had an average of 2281 ng/g less zinc in their liver, compared to controls. Cr(VI) exposure also resulted in significantly decreased hepatic molybdenum, independent of sex (Figure 6G,H). Specifically, Cr(VI)-exposed guinea pigs had an average of 62.6 ng/g less molybdenum in their liver, compared to controls. Conversely, exposure to Cr(VI) did not alter hepatic cobalt or copper levels in either the pooled (Figure 6I,K) or sex-matched data (Figure 6J,L). However, it is worth noting that females had significantly higher baseline levels of cobalt in their liver, compared to males. Overall, these data indicate Cr(VI) exposure altered hepatic essential metals in guinea pigs, especially in females (Figure 7). Pooled and sex-matched essential metal levels in the liver can be found in Tables S3 and S4.

The findings indicate that exposure to Cr(VI) did not significantly alter blood essential metal levels, when considering pooled data (Table 1); however, when separating males and females, numerous changes in essential metal levels became apparent (Table 2; Figure 8). In females only, exposure to Cr(VI) resulted in significant increases in blood iron, copper and zinc and a significant decrease in sodium, compared to control females. For example, in female guinea pigs, Cr(VI) exposure increased the average zinc levels in the blood by 400 ng/L compared to controls. Cr(VI) exposure did not alter blood levels of cobalt or molybdenum in either pooled or sex-matched data (Tables 1 and 2). Independent of exposure, baseline levels of essential metals varied among the sexes. For example, females had significantly lower baseline levels of molybdenum and iron, compared to males. These data further support the sex-specific effects in essential metal dyshomeostasis after exposure to Cr(VI), especially with copper, where opposite effects were observed between sexes in the liver and blood, and the direction of these changes were the same across these tissues.

While chromium only accumulated in the dorsal hippocampus and the pituitary gland, essential metal levels were measured in all 11 brain regions (Table 3). Overall, Cr(VI) exposure significantly decreased levels of five essential metals (potassium, iron, copper, molybdenum and cobalt) across all regions except the ventral hippocampus and pituitary gland (Table 3). For example, in Cr(VI)-exposed guinea pigs, iron levels decreased in the hypothalamus and pituitary gland, and potassium levels decreased in the ventral hippocampus. Most notably, Cr(VI) exposure resulted in significantly decreased cobalt levels in five of the 11 brain regions measured (amygdala, brain stem, cerebellum, entorhinal cortex and striatum) and significantly decreased molybdenum levels in eight of the 11 brain regions measured (amygdala, brain stem, cerebellum, entorhinal cortex, frontal cortex, hypothalamus, olfactory bulb and striatum). When considering sex differences, it became clear that the Cr(VI)-induced decreases of molybdenum and cobalt across various brain regions were driven by the male guinea pigs, as no significant changes in either cobalt or molybdenum levels were seen in any brain region in the females (Table 4, Figure 9). Conversely, Cr(VI)-exposed females had significantly altered levels of sodium, potassium and magnesium, a phenomenon not observed in the males. Specifically, Cr(VI) exposure in female guinea pigs resulted in decreased potassium levels in the dorsal hippocampus, and decreased magnesium levels in the cerebellum, while increased sodium levels were found in the entorhinal and frontal cortices (Table 4, Figure 9B). Independent of exposure, baseline levels of essential metals across the brain regions varied among the sexes. For example, control females had significantly lower baseline levels of molybdenum, magnesium, manganese, and cobalt in their pituitary glands, compared to control males. Overall, these data show both region- and sex-specific alterations in essential metal levels after Cr(VI) exposure.

## 4. Discussion

We developed a novel in vivo model to elucidate the impact of Cr(VI) exposure via drinking water on essential metal levels in the liver, blood and brain. A common denominator between the etiology of diseases in the liver and brain is dyshomeostasis of essential metal [40,41], which Cr(VI) is known to induce across various models and exposure routes [17–19]. Our study reports chromium accumulation in the liver, blood and brain of guinea pigs after subchronic exposure to 5 mg Cr(VI)/L in drinking water, as well as sex-specific essential metal dyshomeostasis in the liver and blood, and sex- and region-specific essential metal dyshomeostasis in the brain.

Our data showed significant chromium accumulation in the livers of guinea pigs after subchronic exposure to Cr(VI) in drinking water. The liver is a target organ of Cr(VI) after drinking-water exposure [6,11], which is consistent with our findings. A previous study in Fisher rats reported significant accumulation of chromium in the livers of rats given 3 or 10 mg Cr(VI)/L in drinking water for 44 weeks, with female rats exhibiting significantly higher hepatic chromium levels than males [50]. By comparison, we observed significant chromium accumulation in the livers of guinea pigs given 5 mg Cr(VI)/L in their drinking water, with significantly higher hepatic chromium levels observed in female guinea pigs. In another study, significant chromium accumulation was reported in guinea pig livers after exposure to particulate Cr(VI) via oropharyngeal aspiration; however, sex-specific chromium accumulation depended on exposure time, with acutely exposed (24 h) females accumulating more hepatic chromium than males, and subchronically exposed (90 days) males accumulating more hepatic chromium than females [18]. It is important to note that the route of exposure for that guinea pig study was oropharyngeal aspiration, not drinking water, which may have contributed to the differences in chromium levels observed in the livers of guinea pigs between these two studies. Specifically, after exposure to Cr(VI) via oropharyngeal aspiration, the first-pass effect of the liver is bypassed.

Chromium levels were significantly elevated in the whole blood of exposed guinea pigs. Red blood cells have a high capacity for Cr(VI) uptake, with red blood cell chromium levels often used as a measure of recent Cr(VI) exposure, due to the short lifespan of red blood cells, which is about 120 days [51,52]. Our data showed female guinea pigs had higher blood chromium levels than males after subchronic exposure to 5 mg Cr(VI)/L. Reported sex differences in chromium accumulation in the blood include a study that quantified Cr(VI) levels in occupationally exposed individuals [53]. This study reported males had higher blood chromium levels when compared to females exposed to the same levels of Cr(VI). However, only 50 of the 195 individuals (26%) included in this study were female. Therefore, this study may not be the best reflection of sex differences in blood chromium levels. Alternatively, Meaza et al. [18] did not find any significant increase in blood chromium levels in either sex after acute or subchronic exposure to particulate Cr(VI) via oropharyngeal aspiration.

While the underlying causes of sex differences in chromium accumulation in the liver, blood, and brain are unclear, one hypothesis is the toxicokinetics of Cr(VI), particularly the absorption through the gastrointestinal tract and distribution to peripheral tissues. The majority of drinking-water Cr(VI) absorption occurs in the villi lining the lumen of the small intestine [54–56]. The absorption of Cr(VI) from the small intestine is largely dependent on pH, with an acidic intestinal environment favoring increased absorption of Cr(VI). The pH of the small intestine in female populations tends to be lower than that of males [57,58]. It is possible the small intestine of female guinea pigs was more acidic than that of male guinea pigs, allowing for increased absorption of Cr(VI) and uptake into tissues. This would explain the significantly increased chromium levels in the livers and blood of exposed female guinea pigs, when compared to males.

Significant chromium accumulation was also seen in the dorsal hippocampi and pituitary glands of female guinea pigs. To our knowledge, chromium accumulation has been reported in the hippocampus of guinea pigs one other time, and that report also noted significant chromium accumulation in the hippocampus of female guinea pigs [19]. While that reported instance was after oropharyngeal aspiration, not drinking-water exposure to Cr(VI), these data support the determination that chromium consistently accumulated in the hippocampus independent of exposure route. There are very few studies that have quantified chromium levels after drinking-water exposure in the brain of rats or mice, most of which only report whole-brain chromium levels and do not consider regional chromium deposition [59]. One study from our collaborators quantified chromium levels in six different brain regions and only found significant chromium accumulation in the hippocampus of female Sprague–Dawley rats following a 90-day exposure to 0.05 or 0.1 mg Cr(VI)/L in drinking water [17]. These data are in line with our observation that chromium significantly accumulated in the hippocampus of female guinea pigs in our study.

Chromium accumulation in the pituitary gland was previously reported in Wistar rats following 30 days of exposure to 100 or 500 mg Cr(VI)/L in drinking water [60,61]. However, these studies were only done in male rats, whereas significant chromium accumulation in the pituitary gland was noted in female guinea pigs in our study. While the cause of this sex difference is unknown, an increase in pituitary gland chromium levels in the female guinea pigs could be directly related to the increased chromium levels in the liver. Estrogen functions as a hepatoprotective agent, reducing the risk of developing chronic liver disease [62,63]. One of the major ways estrogen provides a hepatoprotective effect is by modulating the epithelial cells lining the bile ducts that transport bile to the liver, gallbladder, and small intestine [64–66]. It has been previously reported that chromium can be secreted by the bile as a method of excretion [6]. The pituitary gland produces luteinizing hormone (LH) and follicle-stimulating hormone (FSH), which triggers estrogen production in the ovaries [67]. Chromium accumulation in the pituitary gland of female guinea pigs, as was noted in our study, could decrease the production of LH and FSH, which would reduce estrogen production in the ovaries. This could diminish the hepatoprotective effects of estrogen in the liver, decreasing the flow of bile and reducing the ability of the liver to excrete chromium into the bile. Chromium would then be able to accumulate in the liver tissue of female guinea pigs, as was also observed in this study. However, it is important to note that significant chromium accumulation was also noted in the liver tissue of male guinea pigs in the absence of increased pituitary gland chromium levels. The accumulation of chromium could also be explained by the anatomical position of the pituitary gland. The pituitary gland sits outside of the blood–brain barrier which functions to keep toxicants out of the brain [68], therefore making the pituitary gland more vulnerable to exposure to harmful substances. Overall, the data presented verify previous data showing that chromium can reach the brain and accumulate in a sex- and region-dependent manner by targeting the dorsal hippocampi and pituitary glands of female guinea pigs.

This is the first study to show exposure to Cr(VI) in drinking water causes essential metal dyshomeostasis in the liver, blood and brain of subchronically exposed guinea pigs. Many of these essential metal changes were sex specific. In female guinea pigs only, an interesting observation was seen where essential metal levels tended to decrease in the liver but generally increase in the blood. This effect was particularly striking with zinc, which was significantly decreased in the livers but significantly increased in the blood of female guinea pigs exposed to 5 mg Cr(VI)/L in their drinking water. While the specific cause of these opposing trends is unknown, one possibility is that the decrease in hepatic zinc is attributed to the release of zinc that occurs with hepatocyte injury, allowing zinc to enter circulation and increase blood zinc levels. Essential metal changes in the brain were both region- and sex-specific, with molybdenum and cobalt being significantly decreased in multiple regions in the brains of male, but not female, Cr(VI)-exposed guinea pigs. Disturbances to the essential metal levels that were significantly altered in the liver and brain of guinea pigs have been implicated in contributing to the pathogenesis of disease in both organs.

For example, magnesium deficiency is historically associated with chronic liver diseases. Previous animal studies report that hepatic magnesium deficiencies are associated with increased pro-inflammatory cytokines and morphological changes to liver tissue at the cellular level [69–72]. In the present study, hepatic magnesium levels were significantly decreased after Cr(VI) exposure. When considering the data by sex, only the female guinea pigs had a significant decrease in magnesium levels in the liver. A previous study investigating the impact of particulate Cr(VI) exposure through oropharyngeal aspiration in guinea pigs also showed decreased hepatic magnesium levels in acutely exposed females; however, they also reported acutely exposed male guinea pigs had increased levels of magnesium in their livers, something not observed in the present study [18]. Interestingly, after being subchronically exposed for 90 days, the hepatic magnesium levels returned to baseline. These opposing results in the males could be due to exposure route differences (drinking water vs. oropharyngeal aspiration), Cr(VI) compound differences (soluble vs. particulate), exposure duration (acute vs. subchronic) and/or age differences (~1 year old vs. ~6 months old).

Interestingly, similar to what was observed in the liver, magnesium levels were significantly reduced in the cerebella of Cr(VI)-exposed female guinea pigs, but not males. The cerebellum, a brain region impacted by liver disease [73], is the region of the brain that regulates movement, balance and motor learning [74]. While not well understood, decreased magnesium in the brain can manifest as various neurological conditions, such as Alzheimer’s disease, which females are at a greater risk of developing [75–77]. Notably, the cerebellum was the only region out of the 11 brain regions measured to have altered magnesium levels. To our knowledge, this is the first study to report cerebellum-specific decreases in magnesium levels after Cr(VI) exposure. However, Cr(VI)-induced magnesium deficiency has been reported in the hippocampi of young (6-month-old) female Sprague–Dawley rats after 90 days of exposure via drinking water (essential metal measurements were not reported in the cerebellum) [17]. Together, these data indicate Cr(VI) exposure causes reduced magnesium in the livers and cerebella of female guinea pigs, which may be an early indicator of negative health outcomes in both organs and a potential target when considering organ crosstalk.

Despite the fact that zinc is essential for human health, serving as a cofactor for hundreds of enzymes, few studies have considered the impact of Cr(VI)-exposure on hepatic zinc levels. This is remarkable given that decreased hepatic zinc levels have been associated with the development of chronic liver diseases and cancers for decades [78]. Several studies have reported that the zinc content of cancerous liver tissue is lower than that of surrounding healthy liver tissue [79–84]. In the present study, we found exposure to Cr(VI) significantly decreased hepatic zinc levels. Conversely, those studies that have assessed hepatic zinc levels after Cr(VI) exposure report either no change or increased hepatic zinc [18,85]. There are, however, major differences between the studies that may account for the observed discrepancies in hepatic zinc levels. For example, the study that reported that Cr(VI) exposure induced an increase in hepatic zinc delivered Cr(VI) via a single intraperitoneal injection of soluble potassium dichromate, and animals were euthanized 24 h later [85].

Of the few studies that have assessed hepatic zinc levels after Cr(VI) exposure, even fewer have considered sex as a biological variable, with most studies using only males in their model systems [86–88]. As for those Cr(VI) studies that have considered sex, they either report no differences, or they did not measure hepatic zinc [17,18]. In our study, Cr(VI)-induced decreases of hepatic zinc were largely driven by female guinea pigs, similar to what we observed with hepatic magnesium levels. In the blood, Cr(VI) exposure resulted in significantly increased zinc levels in female guinea pigs only. Notably, these sex-dependent effects appear to be unique to the liver and the blood, as there were no significant changes in zinc levels in any of the isolated brain regions of female or male guinea pigs.

Similar to what was seen with magnesium and zinc, we observed significantly decreased potassium levels in the livers of Cr(VI)-exposed guinea pigs. Low potassium levels are prevalent in people with chronic liver disease; thus, the observed decreased hepatic potassium levels may be an early indicator of liver disease [89–91]. Another study in guinea pigs exposed to particulate Cr(VI) via oropharyngeal aspiration reported decreased hepatic potassium levels after a 24 h acute exposure [17]. However, in this same study, hepatic potassium levels returned to control levels after a subchronic 90-day exposure. Notably, the potassium levels in the livers of control animals from both studies were comparable, and thus there is not an explanation as to why after 90 days we observed decreased hepatic potassium, but the previous study did not.

Potassium was also significantly decreased in the dorsal hippocampi of female guinea pigs. In the brain, potassium plays a key role in maintaining the membrane potential of neurons and subsequent neuronal excitability [91]. One study found that decreased potassium levels in the hippocampi of guinea pigs resulted in epileptiform activity [92] but did not specify the sex of the guinea pigs. In humans, a decrease in hippocampal potassium levels is commonly seen in postmortem tissue from individuals who suffered from vascular dementia [93]. However, most neurodegenerative outcomes relating to potassium in the brain are a result of increased, not decreased, resting potassium levels, as seen in rodent models of Alzheimer’s disease and Huntington’s disease [94,95].

Molybdenum significantly decreased in the livers of Cr(VI)-exposed guinea pigs, but unlike the previous metals, this phenomenon was not associated with a specific sex. Like zinc, molybdenum is a cofactor in a variety of enzymes and is involved with maintaining metabolic homeostasis [96]. However, decreased function of molybdenum-containing enzymes is attributed to a serious genetic disease called molybdenum cofactor deficiency and is not thought to be caused by molybdenum dyshomeostasis [97,98]. Additionally, there are no available data suggesting a connection between low hepatic molybdenum levels and liver disease. In fact, our study is the first to report hepatic molybdenum levels after Cr(VI) exposure and thus warrants further investigation.

While the implications of decreased molybdenum in the liver are currently not well known, the same cannot be said of the brain. Of the 11 brain regions analyzed, molybdenum was significantly decreased in the amygdala, brain stem, entorhinal cortex, hypothalamus, olfactory bulb and striatum of Cr(VI)-exposed male guinea pigs. In our study this was the first outcome that was observed in male, but not female guinea pigs. Molybdenum deficiencies in the brain are most often associated with disorders that impair movement and mobility. When considering sex differences, disorders such as motor neuron disease and Parkinson’s disease are more commonly diagnosed in males [99,100]. There is also a known correlation between Cr(VI) exposure and motor neuron disease, with a study out of Spain reporting a 15.7% increase in motor neuron disease-related deaths in chromium-polluted areas [101].

Limitations: The goal of this pilot study was to begin to establish guinea pigs as a novel drinking-water model for Cr(VI) exposure; therefore we began with one exposure group. Future studies will consider additional Cr(VI) exposure groups to establish a dose response. Further, as a direct result of this study, we will be able to perform a more accurate power analysis to help determine the appropriate number of guinea pigs for each exposure group in future studies, something that was not possible before the data obtained from this study. Also, retired breeders were used for this pilot study. While the cause of the attrition of the three control animals upon arrival was unclear, the fact that they were older, retired breeders may have been a factor. Younger, naive guinea pigs will be considered in future studies, now that it has been shown that guinea pigs can be used as a model for studying exposure to Cr(VI) in drinking water. Regarding the water consumption data, we acknowledge that the precise matching of actual Cr(VI) intake may have been affected by the behavior of male guinea pigs playing with their water bottles, leading to an overestimation of water intake. Thus, future studies will consider different water delivery options to reduce this potential impact. Lastly, it is not possible to distinguish between chromium species (i.e., toxic Cr(VI) and non-toxic Cr(III)) using ICP-MS analysis. Therefore, future studies should include speciation analysis to determine the ratio of these species within guinea pig tissues.

## 5. Conclusions

Our study is the first to report that subchronic exposure to 5 mg Cr(VI)/L in drinking water causes sex-dependent essential metal dyshomeostasis in the liver and blood, and sex- and region-dependent essential metal dyshomeostasis in the brains of guinea pigs. Our study shows significant accumulation of chromium in the liver, blood and brain of guinea pigs, particularly in the females. Female guinea pigs were more sensitive to Cr(VI)-induced hepatic essential metal dyshomeostasis, as seen in the significantly decreased magnesium, zinc and potassium levels in the liver. Male guinea pigs were more vulnerable to Cr(VI)-induced essential metal dyshomeostasis of the brain, as evidenced by the significant decrease in molybdenum in several brain regions. Taken together, using a novel in vivo model, these data provide new insight into the sex-specific effects of exposure to Cr(VI) in drinking water on essential metal dyshomeostasis in the liver, blood and brain of guinea pigs. These findings highlight the need for future studies investigating the impact of Cr(VI)-induced essential metals dyshomeostasis on liver and brain health as well as the sex-dependent differences in adverse health outcomes. Importantly, assessment of liver dysfunction, neurodegeneration and behavioral changes are warranted in future studies in order to correlate metals dyshomeostasis with negative health outcomes.

## Supplementary Material

Supp Material

**Supplementary Materials:** The following supporting information can be downloaded at: https://www.mdpi.com/article/10.3390/livers6050090/s1, Table S1. Quality assurance and quality control analysis of liver and blood samples analyzed on an Agilent 7900 ICP-MS by the Integrative Molecular Analysis Core at the University of New Mexico. Table S2. Quality assurance and quality control analysis of brain samples analyzed on an Agilent 7900 ICP-MS by the Metallomics Core at the University of Louisville. Table S3. Pooled essential metal levels in the liver. Table S4. Sex-matched essential metal levels in the liver. Figure S1. Chromium did not significantly accumulate in other isolated brain regions.

## Figures and Tables

**Figure 1.**
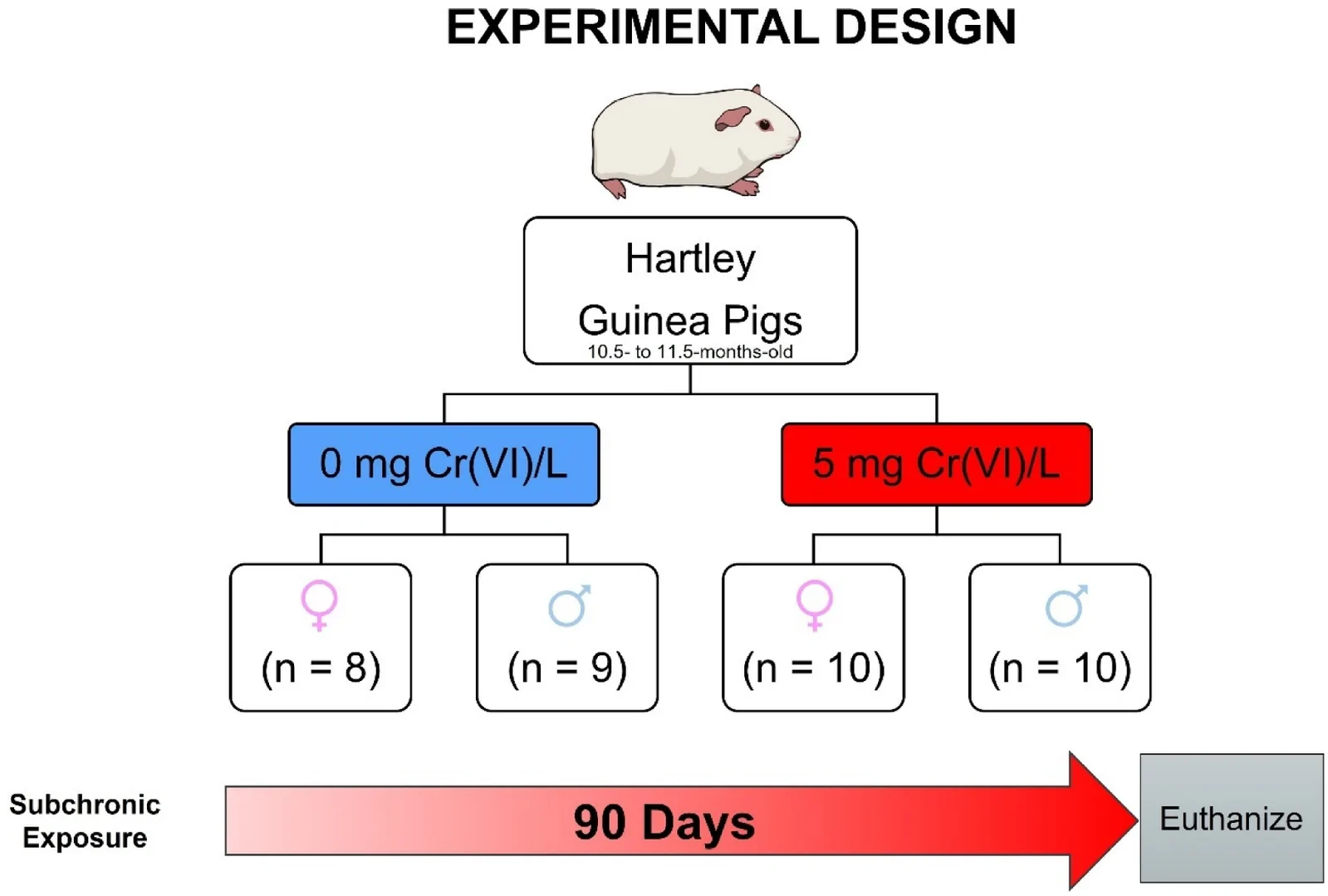
Experimental design and exposure paradigm. Male (*n* = 19) and female (*n* = 18) Hartley guinea pigs, aged 10.5–11.5 months old, were given tap water (control) or 5 mg Cr(VI)/mL in tap water for 90 days. At the end of 90 days, guinea pigs were euthanized, and liver, blood and brain samples were collected for metals analyses.

**Figure 2.**
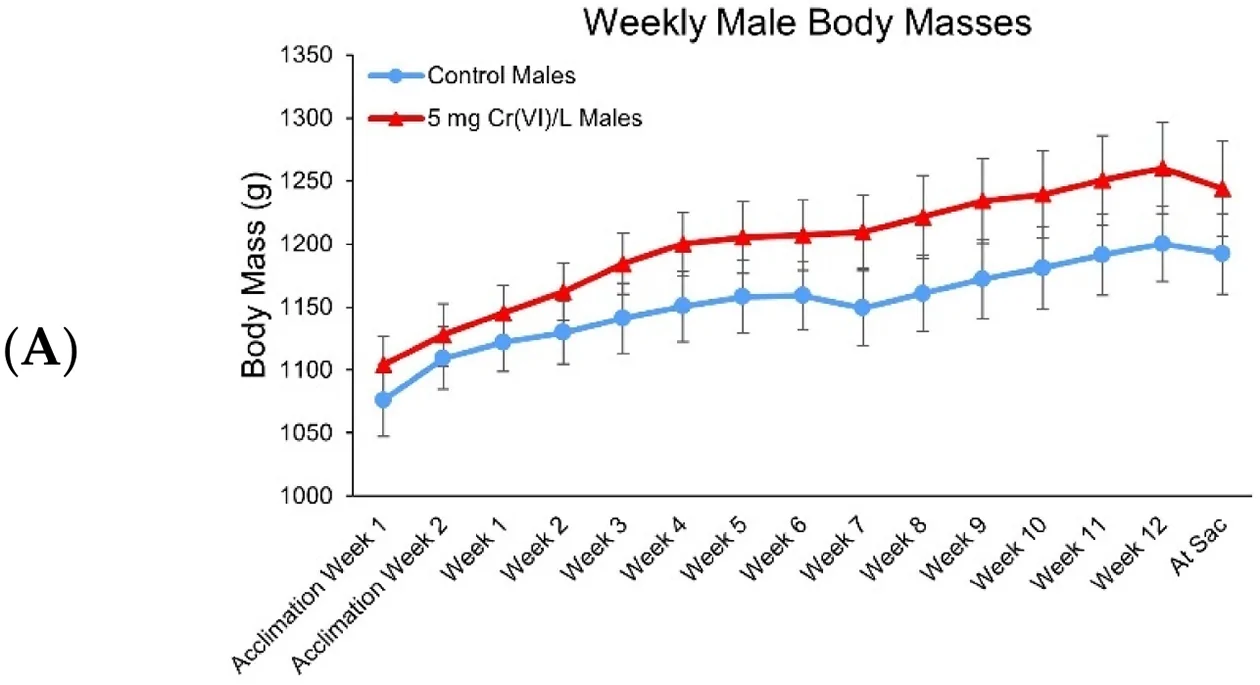

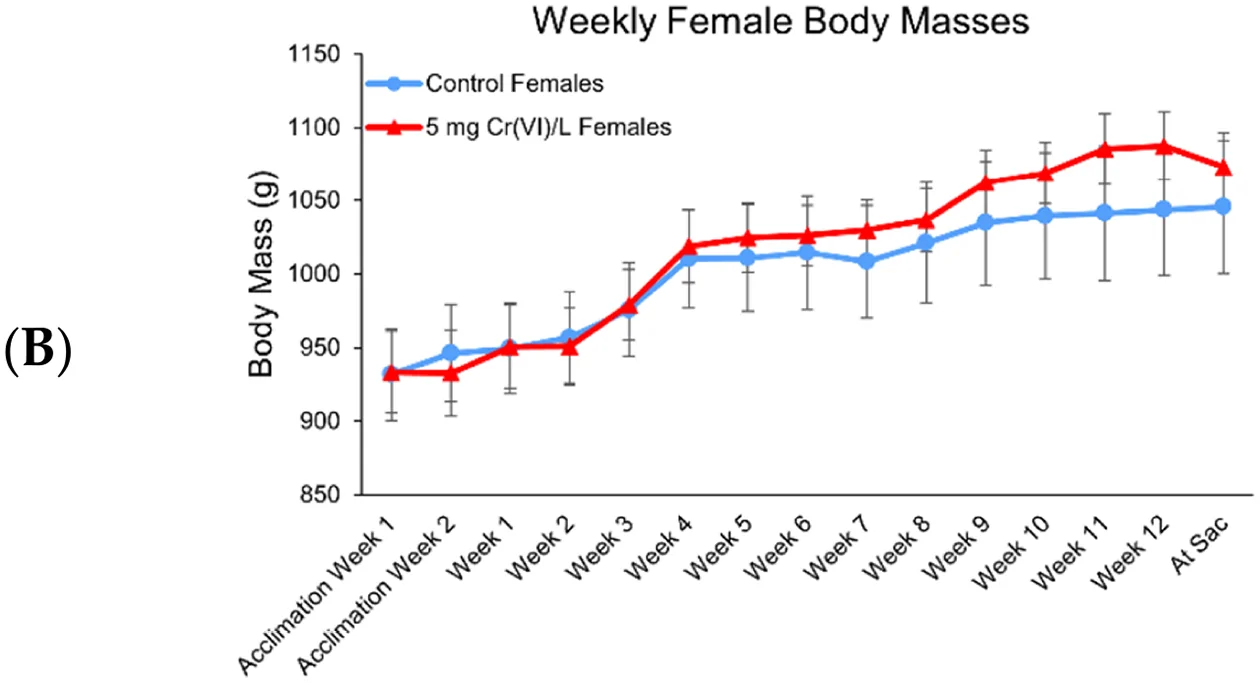
Exposure to Cr(VI) for 90 days increased the whole-body mass of both (**A**) males and (**B**) females, compared to sex-matched controls. Data is reported as mean ± standard error of the mean (*n* = 8–10 per group).

**Figure 3.**
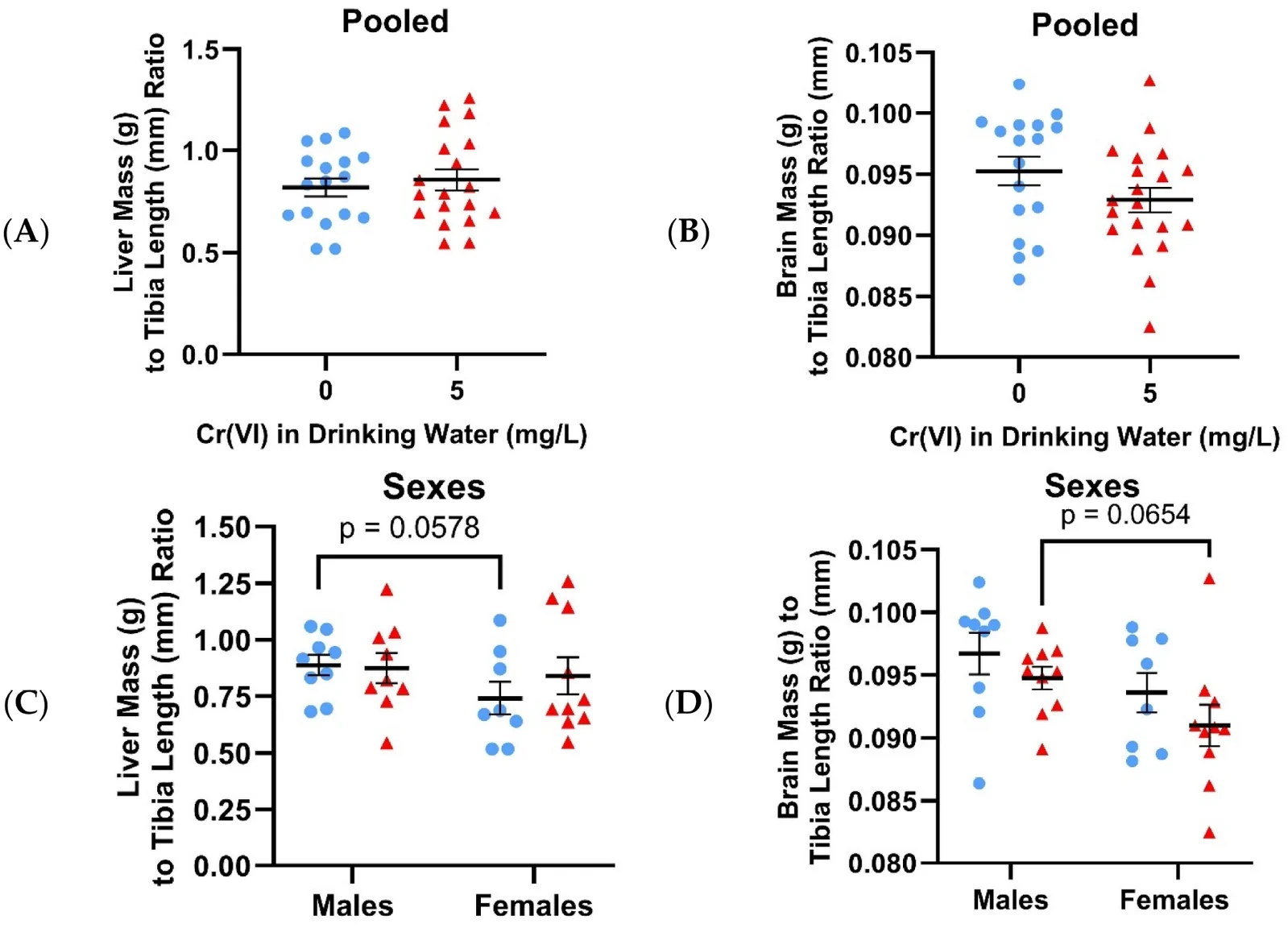
Subchronic exposure to Cr(VI) in drinking water did not alter liver- or brain-to-tibia ratios. (**A**) Liver-mass-to-tibia-length ratio. (**B**) Brain-mass-to-tibia-length ratio. (**C**) Liver-mass-to-tibia-length ratio, separated by sex. (**D**) Brain-mass-to-tibia-length ratio, separated by sex. Each dot represents one animal, with blue circles representing animals in the control group and red triangles representing animals exposed to 5 mg Cr(VI)/L in drinking water. Data is reported as mean ± standard error of the mean (*n* = 18–20 per exposure group and 8–10 per sex).

**Figure 4.**
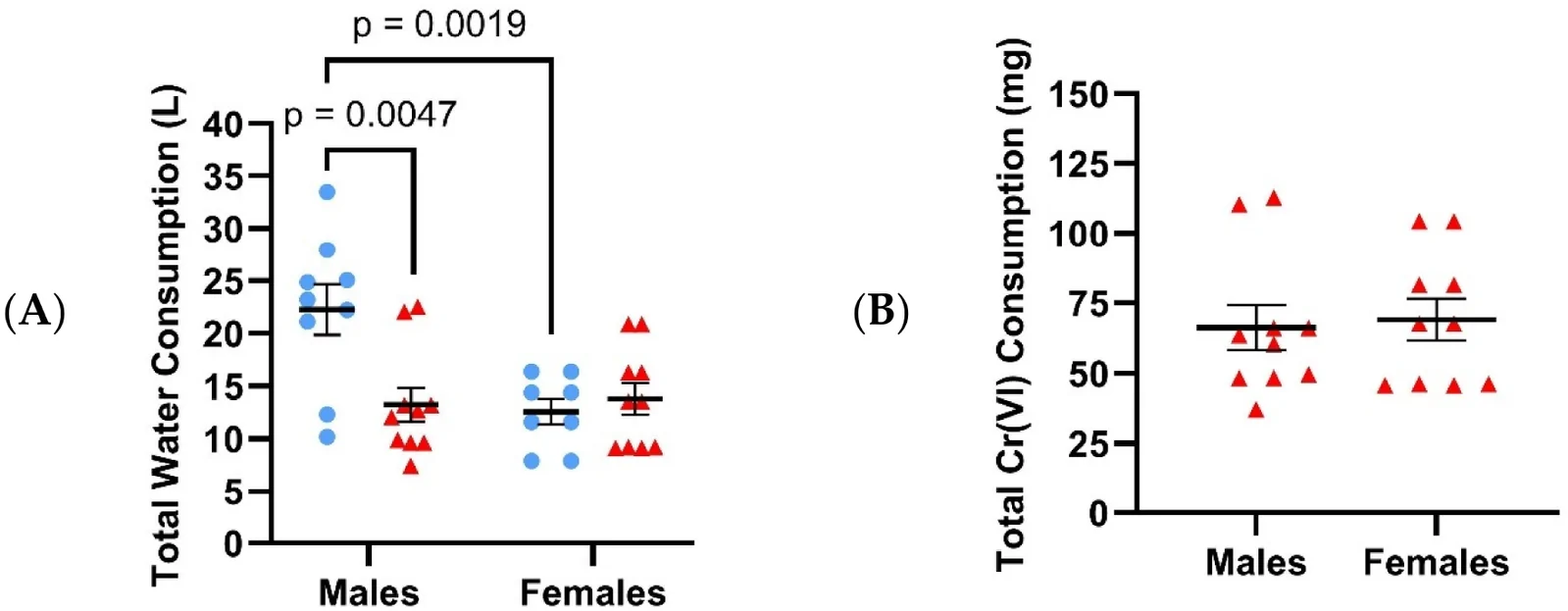
Male and female guinea pigs consumed a similar amount of Cr(VI) from their drinking water. (**A**) Total water consumption, separated by sex. (**B**) Total Cr(VI) consumption, separated by sex. Each dot represents one animal, with blue circles representing animals in the control group and red triangles representing animals exposed to 5 mg Cr(VI)/L in drinking water. Data is reported as mean ± standard error of the mean (*n* = 8–10 per sex).

**Figure 5.**
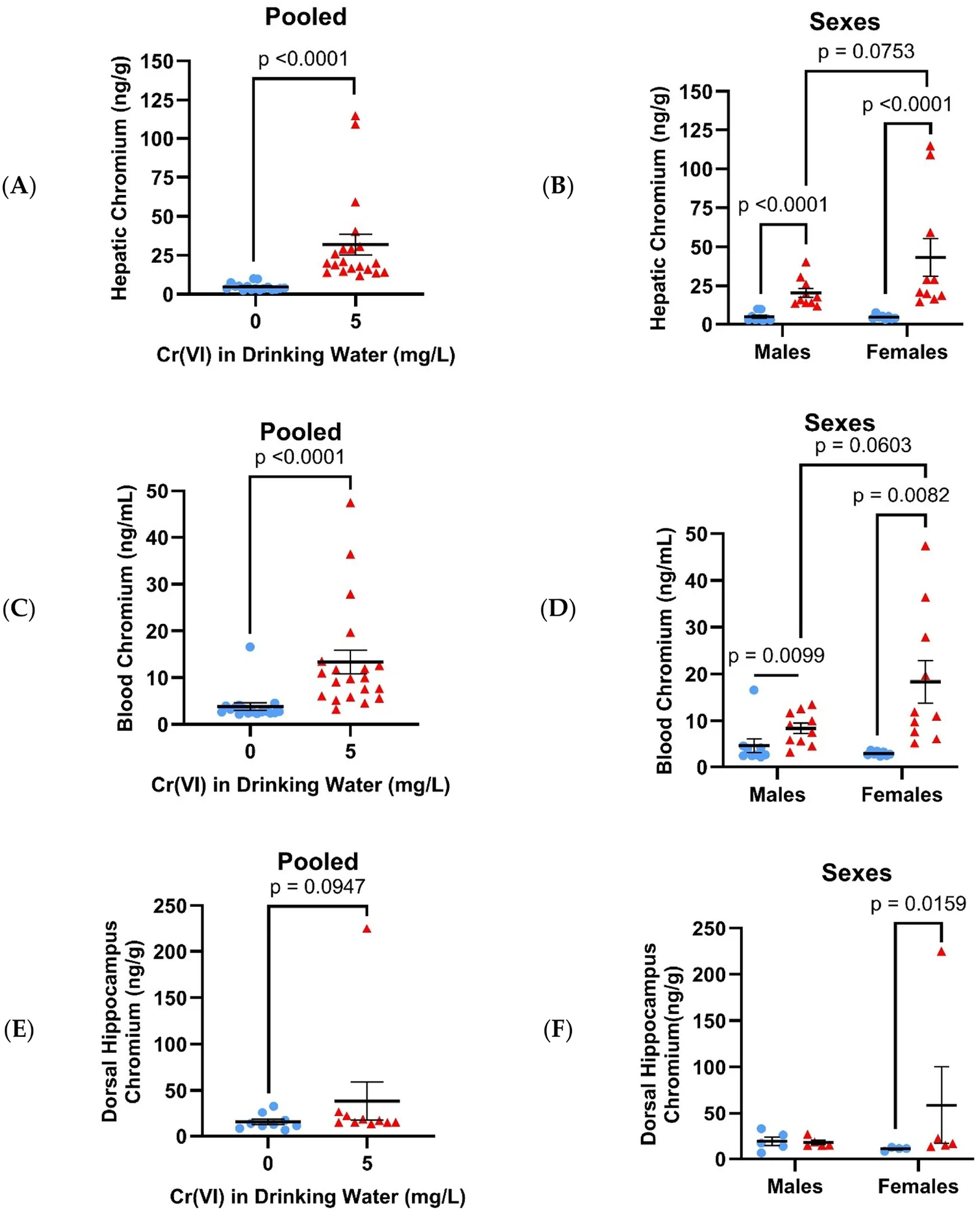

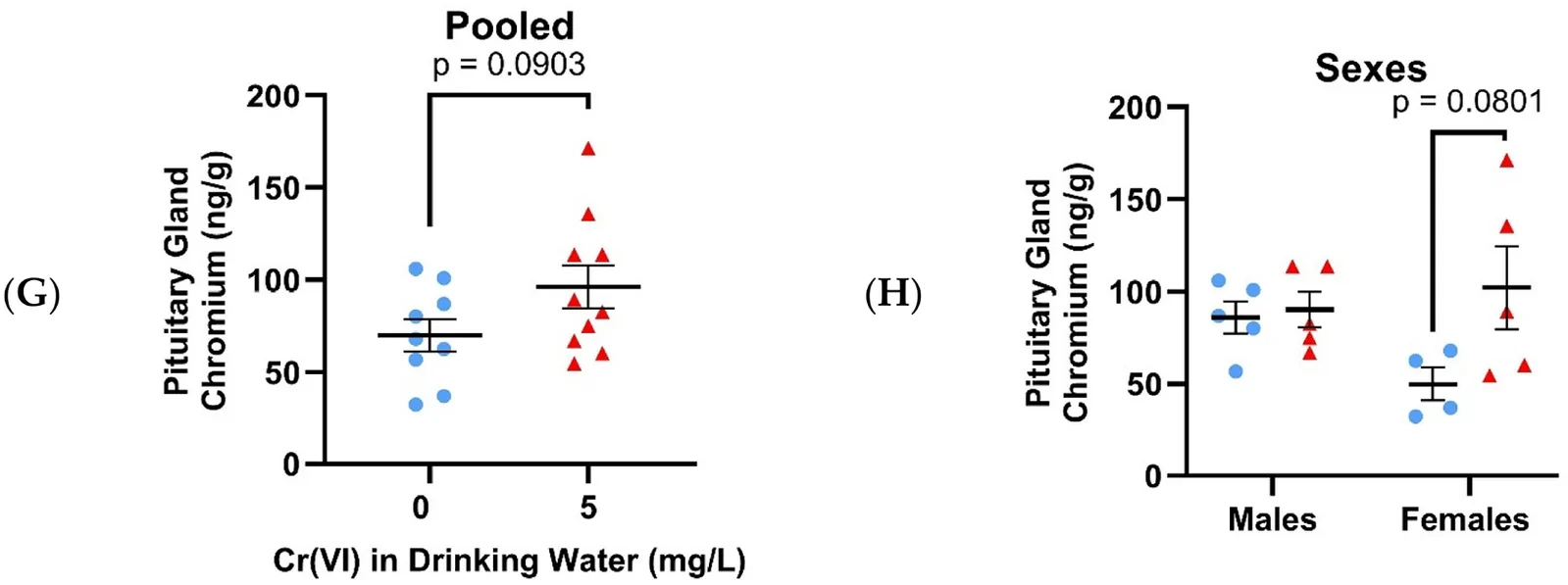
Chromium accumulated in the liver, blood and brain of exposed guinea pigs, with females consistently exhibiting higher levels. (**A**) Hepatic chromium. (**B**) Hepatic chromium, separated by sex. (**C**) Blood chromium. (**D**) Blood chromium, separated by sex. (**E**) Dorsal hippocampus chromium. (**F**) Dorsal hippocampus chromium, separated by sex. (**G**) Pituitary gland chromium. (**H**) Pituitary gland chromium, separated by sex. Each dot represents one animal, with blue circles representing animals in the control group and red triangles representing animals exposed to 5 mg Cr(VI)/L in drinking water. Data is reported as mean ± standard error of the mean (*n* = 18–20 per exposure group and 8–10 per sex for liver and blood; *n* = 9–10 per exposure group and 4–5 per sex for the dorsal hippocampus and pituitary gland).

**Figure 6.**
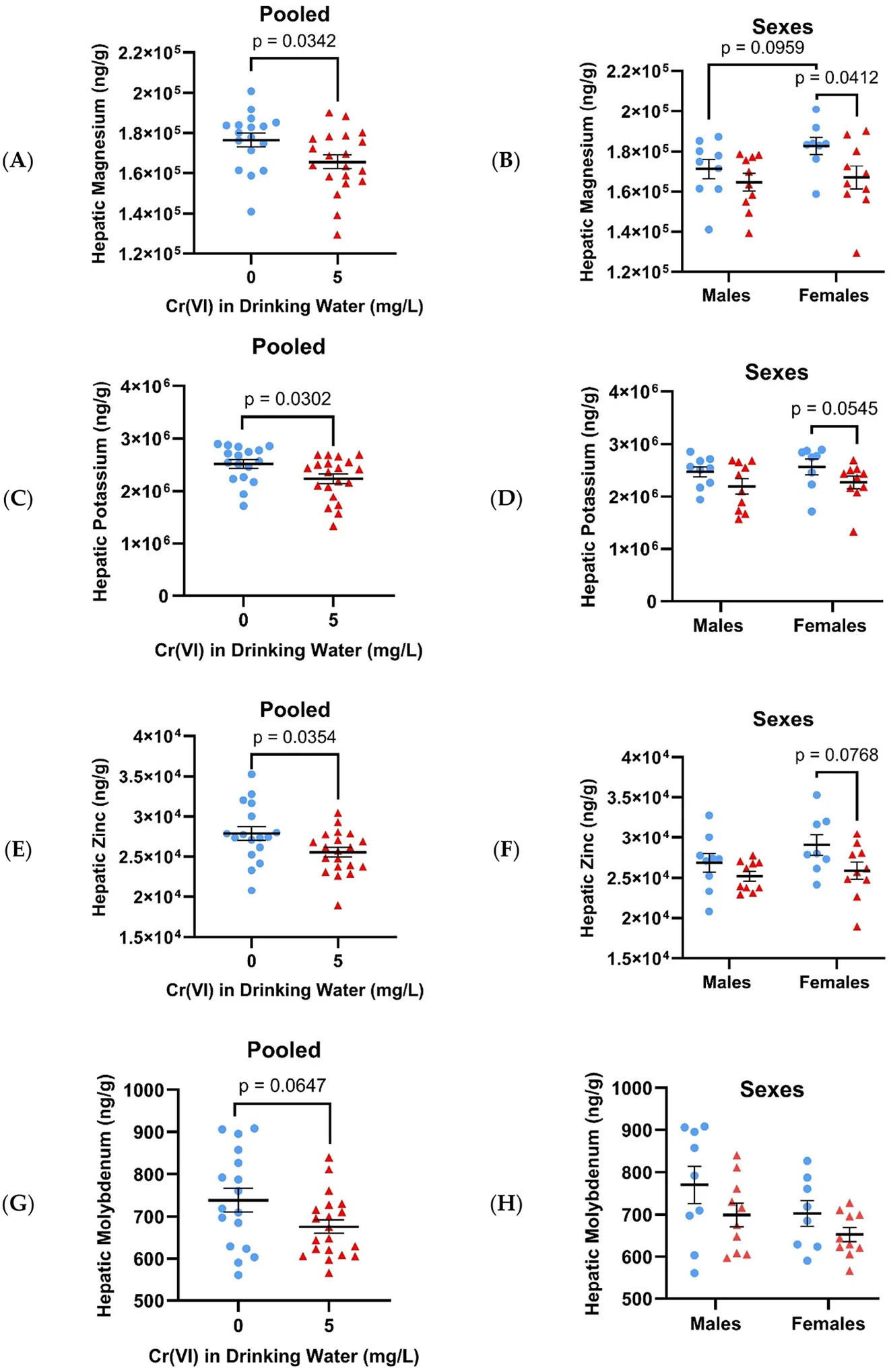

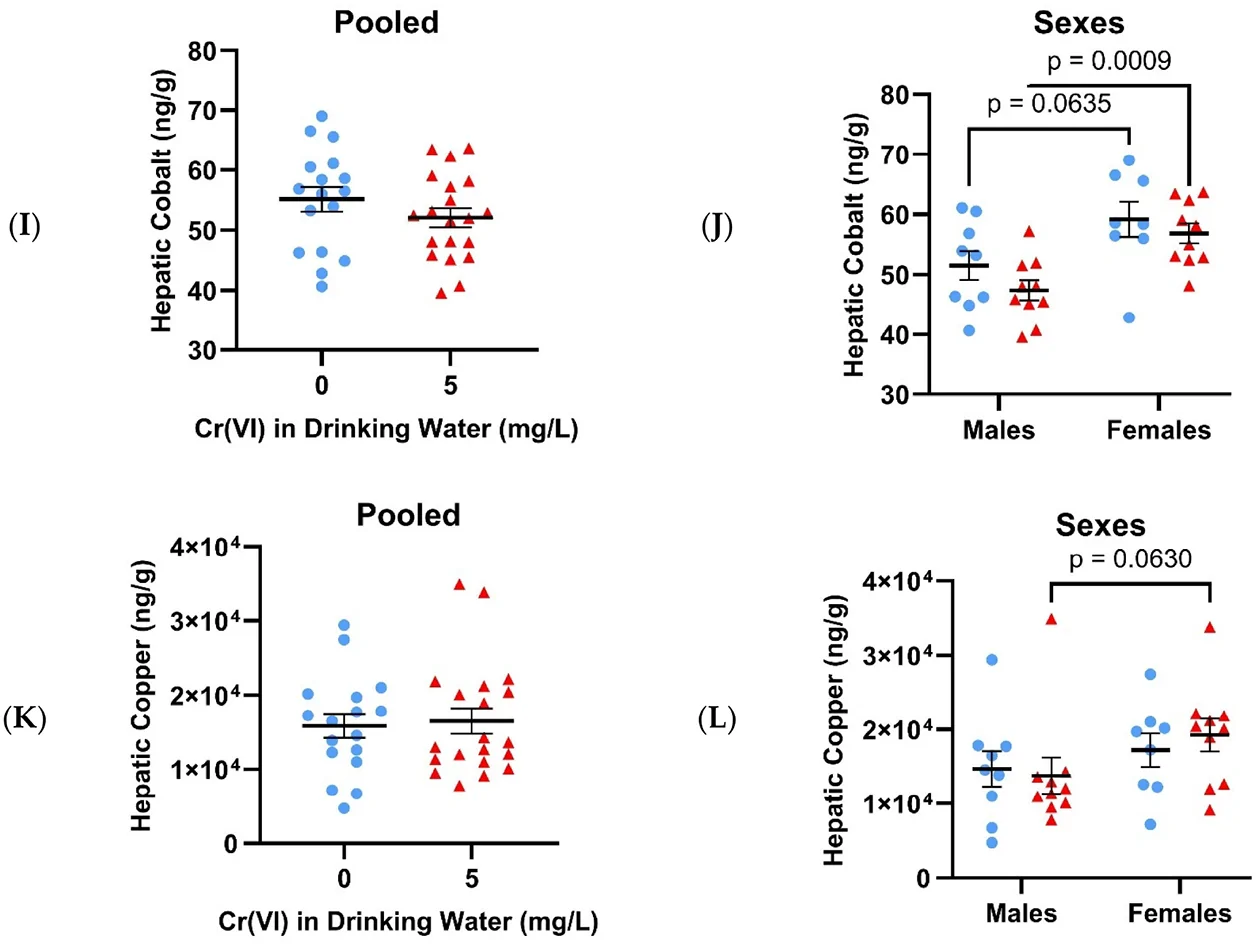
Drinking-water Cr(VI) exposure induced sex-specific essential metal dyshomeostasis in the liver of guinea pigs. (**A**) Hepatic magnesium. (**B**) Hepatic magnesium, separated by sex. (**C**) Hepatic potassium. (**D**) Hepatic potassium, separated by sex. (**E**) Hepatic zinc. (**F**) Hepatic zinc, separated by sex. (**G**) Hepatic molybdenum. (**H**) Hepatic molybdenum, separated by sex. (**I**) Hepatic cobalt. (**J**) Hepatic cobalt, separated by sex. (**K**) Hepatic copper. (**L**) Hepatic copper, separated by sex. Each dot represents one animal, with blue circles representing animals in the control group and red triangles representing animals exposed to 5 mg Cr(VI)/L in drinking water. Data is reported as mean ± standard error of the mean (*n* = 18–20 per exposure group and 8–10 per sex).

**Figure 7.**
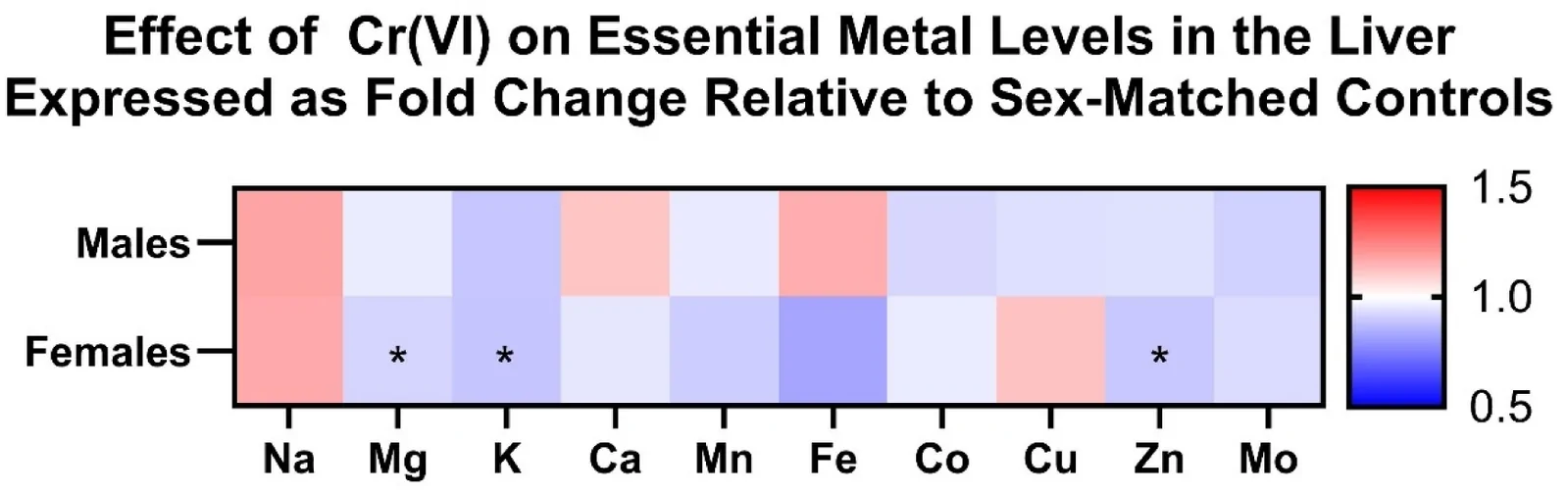
Exposure to Cr(VI) induced sex-dependent essential metal dyshomeostasis in the liver of guinea pigs. The red boxes indicate essential metal levels that were increased compared to sex-matched controls, while the blue boxes indicate essential metal levels that were decreased compared to sex-matched controls. (*) *p* < 0.1 compared to sex-matched controls. Na = sodium; Mg = magnesium; K = potassium; Ca = calcium; Mn = manganese; Fe = iron; Co = cobalt; Cu = copper; Zn = zinc; Mo = molybdenum.

**Figure 8.**
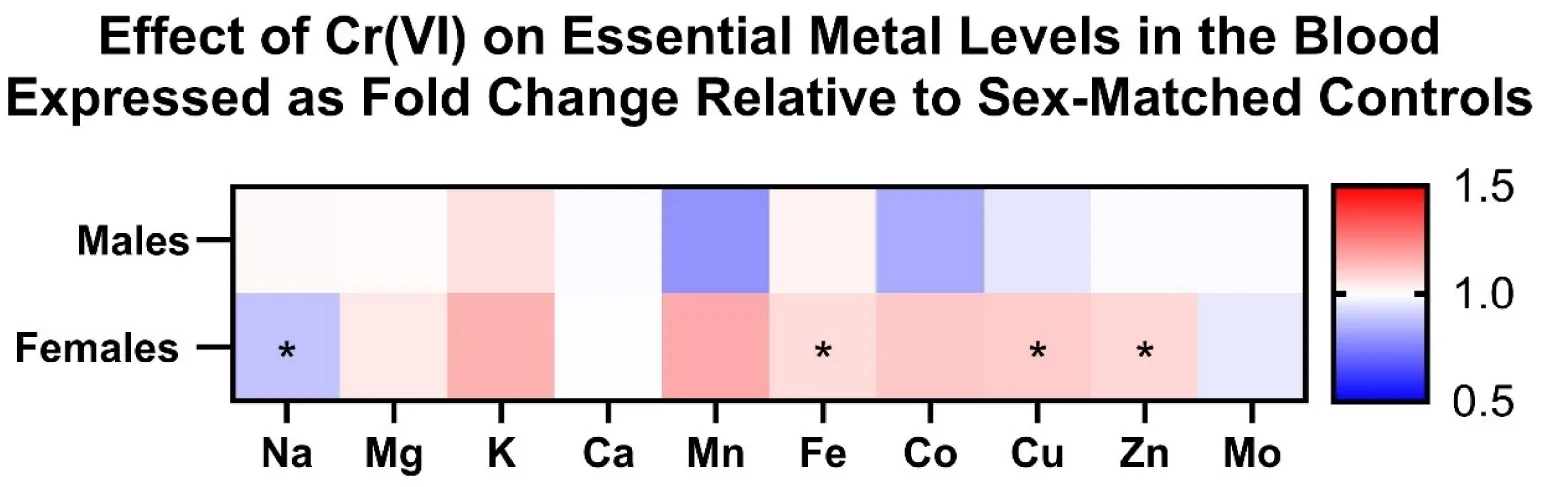
Exposure to Cr(VI) induced sex-dependent essential metal dyshomeostasis in the blood of guinea pigs. The red boxes indicate essential metal levels that were increased compared to sex-matched controls, while the blue boxes indicate essential metal levels that were decreased compared to sex-matched controls. (*) *p* < 0.1 compared to sex-matched controls. Na = sodium; Mg = magnesium; K = potassium; Ca = calcium; Mn = manganese; Fe = iron; Co = cobalt; Cu = copper; Zn = zinc; Mo = molybdenum.

**Figure 9.**
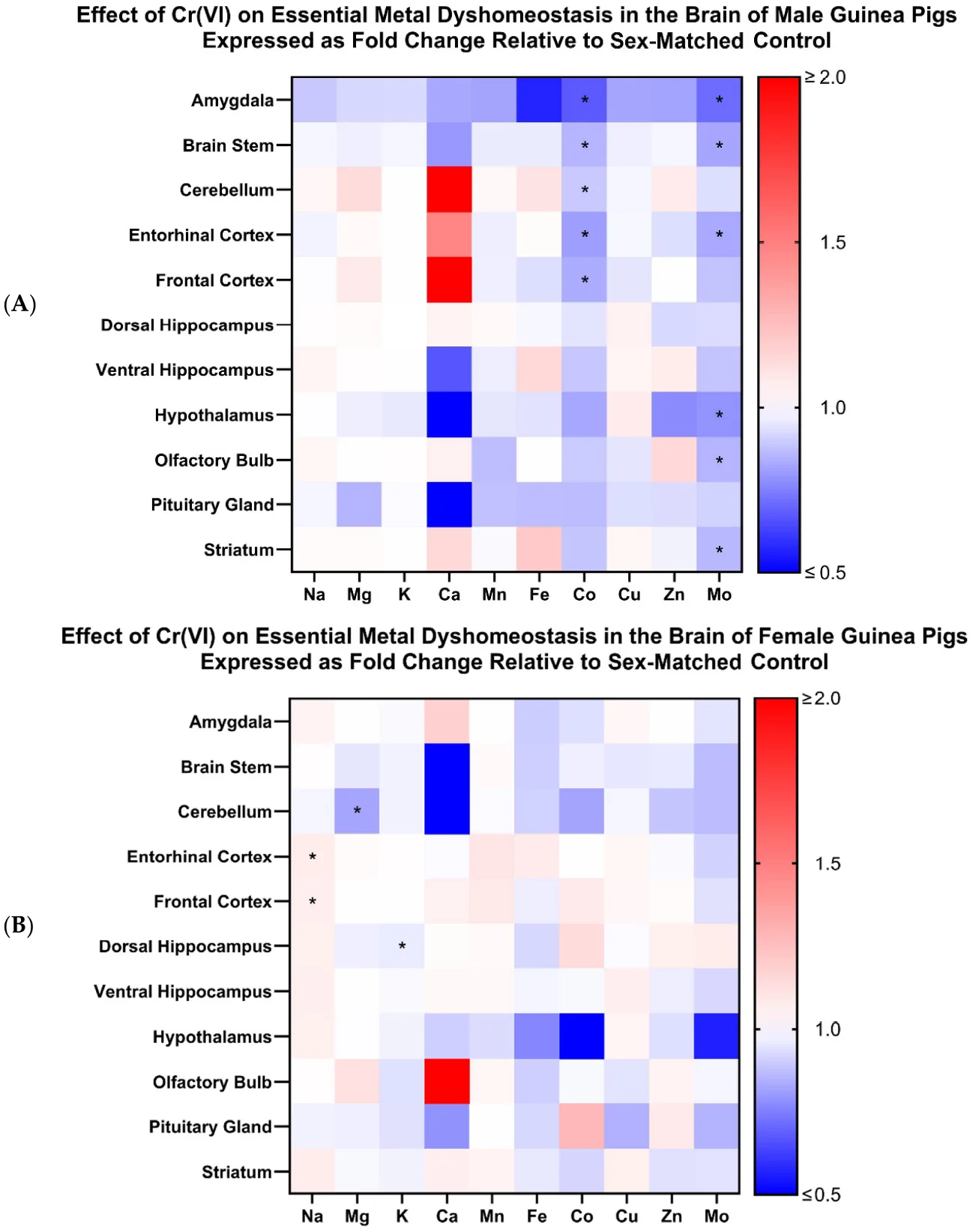
Exposure to Cr(VI) induced sex- and region-dependent essential metal dyshomeostasis in the brain of guinea pigs. (**A**) Overview of essential metal changes seen in isolated brain regions of male guinea pigs. (**B**) Overview of essential metal changes seen in isolated brain regions of female guinea pigs. The red boxes indicate essential metal levels that were increased compared to sex-matched controls, while the blue boxes indicate essential metal levels that were decreased compared to sex-matched controls. (*) *p* < 0.1 compared to sex-matched controls. Na = sodium; Mg = magnesium; K = potassium; Ca = calcium; Mn = manganese; Fe = iron; Co = cobalt; Cu = copper; Zn = zinc; Mo = molybdenum.

**Table 1.** Pooled essential metal levels in the blood.

Exposure Group	Sodium	Magnesium	Potassium	Calcium	Manganese	Iron	Cobalt	Copper	Zinc	Molybdenum
0 mg Cr(VI)/L (*n* = 16–17)	2.3 × 10^6^ ± 9.5 × 10^4^	3.4 × 10^4^ ± 7.1 × 10^2^	2.9 × 10^6^ ± 1.6 × 10^5^	5.4 × 10^4^ ± 1.2 × 10^3^	34 ± 4.8	4.4 × 10^5^ ± 1.1 × 10^4^	0.96 ± 0.080	5.5 × 10^2^ ± 19	4.7 × 10^3^ ± 1.1 × 10^2^	13 ± 0.55
5 mg Cr(VI)/L (*n* = 20)	2.2 × 10^6^ ± 7.7 × 10^4^	3.5 × 10^4^ ± 6.2 × 10^2^	3.2 × 10^6^ ± 1.5 × 10^5^	5.4 × 10^4^ ± 7.5 × 10^2^	32 ± 2.7	4.6 × 10^5^ ± 1.1 × 10^4^	0.91 ± 0.057	5.7 × 10^2^ ± 16	4.9 × 10^3^ ± 86	13 ± 0.53

Data are reported as mean (ng/mL) ± standard error of the mean.

**Table 2.** Essential metal levels in the blood by sex.

Sex	Exposure Group	Sodium	Magnesium	Potassium	Calcium	Manganese	Iron	Cobalt	Copper	Zinc	Molybdenum
Male	0 mg Cr(VI)/L (*n* = 8–9)	2.2 × 10^6^ ± 1.2 × 10^5^	3.5 × 10^4^ ± 9.2 × 10^2^	3.1 × 10^6^ ± 2.5 × 10^5^	5.4 × 10^4^ ± 1.3 × 10^3^	38 ± 8.8	4.7 × 10^5^ ± 1.4 × 10^4 c^	0.97 ± 0.093	5.4 × 10^2^ ± 30	5.0 × 10^3^ ± 1.1 × 10^2 c^	15 ± 0.71^c^
	5 mg Cr(VI)/L (*n* = 10)	2.2 × 10^6^ ± 8.7 × 10^4^	3.6 × 10^4^ ± 9.5 × 10^2^	3.3 × 10^6^ ± 2.3 × 10^5^	5.4 × 10^4^ ± 1.3 × 10^3^	30 ± 1.2	4.8 × 10^5^ ± 1.6 × 10^4^^d^	0.81 ± 0.054^d^	5.1 × 10^2^ ± 15^d^	5.0 × 10^3^ ± 1.3 × 10^2^	14 ± 0.71^d^
Female	0 mg Cr(VI)/L (*n* = 8)	2.5 × 10^6^ ± 1.3 × 10^5^^b^	3.2 × 10^4^ ± 8.2 × 10^2^	2.7 × 10^6^ ± 1.7 × 10^5^	5.4 × 10^4^ ± 2.2 × 10^3^	30 ± 2.9	4.1 × 10^5^ ± 9.2 × 10^3^^b,c^	0.95 ± 0.14	5.7 × 10^2^ ± 24^b^	4.4 × 10^3^ ± 1.2 × 10^2^^b,c^	12 ± 0.64^c^
	5 mg Cr(VI)/L (*n* = 10)	2.2 × 10^6^ ± 1.3 × 10^5^^b^	3.4 × 10^4^ ± 7.2 × 10^2^	3.1 × 10^6^ ± 1.8 × 10^5^	5.4 × 10^4^ ± 7.7 × 10^2^	35 ± 5.3	4.4 × 10^5^ ± 1.3 × 10^4^^b,d^	1.0 ± 0.092^d^	6.2 × 10^2^ ± 16^b,d^	4.8 × 10^3^ ± 1.1 × 10^2^^b^	12 ± 0.50^d^

Data are reported as mean (ng/mL) ± standard error of the mean.

(b) *p* < 0.1 between control females and Cr(VI)-exposed females;

(c) *p* < 0.1 between control males and control females;

(d) *p* < 0.1 between Cr(VI)-exposed males and Cr(VI)-exposed females.

**Table 3.** Pooled essential metal levels in the brain.

Region	Exposure Group	Sodium	Magnesium	Potassium	Calcium	Manganese	Iron	Cobalt	Copper	Zinc	Molybdenum
Amygdala	0 mg Cr(VI)/L (*n* = 9)	1.4 × 10^6^ ± 8.1 × 10^4^	1.8 × 10^5^ ± 8.6 × 10^3^	4.0 × 10^6^ ± 1.6 × 10^5^	1.1 × 10^5^ ± 9.4 × 10^3^	4.0 × 10^2^ ± 31	2.7 × 10^4^ ± 7.0 × 10^3^	**20** ± **1.9** *	4.6 × 10^3^ ± 4.6 × 10^2^	3.1 × 10^4^ ± 2.2 × 10^3^	**32** ± **2.1** *
	5 mg Cr(VI)/L (*n* = 10)	1.3 × 10^6^ ± 2.6 × 10^4^	1.7 × 10^5^ ± 4.0 × 10^3^	3.8 × 10^6^ ± 9.0 × 10^4^	1.0 × 10^5^ ± 8.5 × 10^3^	3.5 × 10^2^ ± 17	1.8 × 10^4^ ± 9.1 × 10^2^	**16** ± **0.91** *	4.1 × 10^3^ ± 2.5 × 10^2^	2.8 × 10^4^ ± 2.3 × 10^3^	**25** ± **1.3** *
Brain Stem	0 mg Cr(VI)/L (*n* = 9)	7.0 × 10^5^ ± 6.5 × 10^3^	8.8 × 10^4^ ± 2.1 × 10^3^	1.7 × 10^6^ ± 1.8 × 10^4^	1.9 × 10^5^ ± 1.2 × 10^5^	2.0 × 10^2^ ± 4.1	1.3 × 10^4^ ± 6.5 × 10^2^	**4.7** ± **0.09** *	1.6 × 10^3^ ± 69	5.0 × 10^3^ ± 87	**14** ± **0.47** *
	5 mg Cr(VI)/L (*n* = 10)	6.9 × 10^5^ ± 7.2 × 10^3^	8.5 × 10^4^ ± 9.2 × 10^2^	1.6 × 10^6^ ± 1.5 × 10^4^	8.1 × 10^4^ ± 2.3 × 10^4^	2.0 × 10^2^ ± 7.7	1.2 × 10^4^ ± 3.5 × 10^2^	**4.2** ± **0.12** *	1.5 × 10^3^ ± 40	4.9 × 10^3^ ± 36	**12** ± **0.58** *
Cerebellum	0 mg Cr(VI)/L (*n* = 9)	7.4 × 10^5^ ± 1.7 × 10^4^	9.6 × 10^4^ ± 6.7 × 10^3^	1.7 × 10^6^ ± 8.4 × 10^3^	5.1 × 10^5^ ± 2.7 × 10^5^	2.6 × 10^2^ ± 6.1	1.2 × 10^4^ ± 3.5 × 10^2^	**6.0** ± **0.56** *	1.8 × 10^3^ ± 74	6.5 × 10^3^ ± 3.1 × 10^2^	**13** ± **0.39** *
	5 mg Cr(VI)/L (*n* = 10)	7.5 × 10^5^ ± 7.9 × 10^3^	9.3 × 10^4^ ± 4.5 × 10^3^	1.7 × 10^6^ ± 1.7 × 10^4^	4.7 × 10^5^ ± 2.4 × 10^5^	2.6 × 10^2^ ± 10	1.2 × 10^4^ ± 6.4 × 10^2^	**5.2** ± **0.10** *	1.8 × 10^3^ ± 56	6.4 × 10^3^ ± 1.7 × 10^2^	**12** ± **0.55** *
Entorhinal Cortex	0 mg Cr(VI)/L (*n* = 9)	7.6 × 10^5^ ± 1.0 × 10^4^	8.3 × 10^4^ ± 1.1 × 10^3^	1.8 × 10^6^ ± 2.3 × 10^4^	3.6 × 10^4^ ± 5.1 × 10^3^	1.5 × 10^2^ ± 4.3	8.9 × 10^3^ ± 3.8 × 10^2^	**5.7** ± **0.15** *	1.4 × 10^3^ ± 53	1.0 × 10^4^ ± 2.2 × 10^2^	**12** ± **0.44** *
	5 mg Cr(VI)/L (*n* = 10)	7.8 × 10^5^ ± 1.4 × 10^4^	8.4 × 10^4^ ± 6.1 × 10^2^	1.8 × 10^6^ ± 1.4 × 10^4^	4.4 × 10^4^ ± 1.1 × 10^4^	1.5 × 10^2^ ± 4.3	9.2 × 10^3^ ± 4.4 × 10^2^	**5.1** ± **0.15** *	1.4 × 10^3^ ± 48	9.7 × 10^3^ ± 2.2 × 10^2^	**10** ± **0.40** *
Frontal Cortex	0 mg Cr(VI)/L (*n* = 9)	6.8 × 10^5^ ± 1.3 × 10^4^	8.5 × 10^4^ ± 8.9 × 10^2^	1.8 × 10^6^ ± 1.8 × 10^4^	9.5 × 10^4^ ± 2.2 × 10^4^	1.5 × 10^2^ ± 5.3	9.4 × 10^3^ ± 2.8 × 10^2^	5.4 ± 0.23	1.3 × 10^3^ ± 62	7.8 × 10^3^ ± 1.4 × 10^2^	**13** ± **0.40** *
	5 mg Cr(VI)/L (*n* = 10)	6.9 × 10^5^ ± 1.2 × 10^4^	8.9 × 10^4^ ± 3.9 × 10^3^	1.8 × 10^6^ ± 1.4 × 10^4^	3.4 × 10^5^ ± 2.3 × 10^5^	1.5 × 10^2^ ± 6.4	8.9 × 10^3^ ± 2.7 × 10^2^	5.1 ± 0.11	1.3 × 10^3^ ± 49	7.8 × 10^3^ ± 2.2 × 10^2^	**11** ± **0.43** *
Dorsal Hippocampus	0 mg Cr(VI)/L (*n* = 9)	1.2 × 10^6^ ± 2.3 × 10^4^	1.5 × 10^5^ ± 1.1 × 10^3^	3.2 × 10^6^ ± 2.7 × 10^4^	5.9 × 10^4^ ± 1.7 × 10^3^	3.2 × 10^2^ ± 7.0	1.9 × 10^4^ ± 2.2 × 10^3^	9.2 ± 0.32	3.0 × 10^3^ ± 1.0 × 10^2^	1.8 × 10^4^ ± 4.6 × 10^2^	22 ± 0.85
	5 mg Cr(VI)/L (*n* = 10)	1.2 × 10^6^ ± 3.1 × 10^4^	1.5 × 10^5^ ± 1.9 × 10^3^	3.1 × 10^6^ ± 4.3 × 10^4^	6.0 × 10^4^ ± 2.0 × 10^3^	3.2 × 10^2^ ± 16	1.8 × 10^4^ ± 1.8 × 10^3^	9.5 ± 0.43	3.1 × 10^3^ ± 89	1.8 × 10^4^ ± 8.9 × 10^2^	22 ± 1.9
Ventral Hippocampus	0 mg Cr(VI)/L (*n* = 9)	1.2 × 10^6^ ± 2.7 × 10^4^	1.4 × 10^5^ ± 4.0 × 10^3^	**3.1** × **10**^**6**^ ± **1.0** × **10**^**5**^ *	7.6 × 10^4^ ± 1.7 × 10^4^	3.3 × 10^2^ ± 14	1.8 × 10^4^ ± 1.3 × 10^3^	10 ± 0.51	3.1 × 10^3^ ± 1.1 × 10^3^	1.7 × 10^4^ ± 8.2 × 10^2^	21 ± 1.3
	5 mg Cr(VI)/L (*n* = 10)	1.2 × 10^6^ ± 1.7 × 10^4^	1.5 × 10^5^ ± 1.1 × 10^3^	**3.1** × **10**^**6**^ ± **2.5** × **10**^**4**^ *	6.0 × 10^4^ ± 2.1 × 10^3^	3.3 × 10^2^ ±12	1.9 × 10^4^ ± 1.8 × 10^3^	9.4 ± 0.21	3.2 × 10^3^ ± 63	1.7 × 10^4^ ± 7.8 × 10^2^	19 ± 0.93
Hypothalamus	0 mg Cr(VI)/L (*n* = 9)	1.3 × 10^6^ ± 4.9 × 10^4^	1.7 × 10^5^ ± 5.3 × 10^3^	3.7 × 10^6^ ± 1.1 × 10^5^	1.4 × 10^5^ ± 6.5 × 10^4^	5.2 × 10^2^ ± 28	**2.7** × **10**^**4**^ ± **2.1** × **10**^**3**^ *	31 ± 16	4.3 × 10^3^ ± 1.5 × 10^2^	1.8 × 10^4^ ± 1.6 × 10^3^	**49** ± **9.8** *
	5 mg Cr(VI)/L (*n* = 10)	1.4 × 10^6^ ± 2.6 × 10^4^	1.6 × 10^5^ ± 2.8 × 10^3^	3.6 × 10^6^ ± 7.2 × 10^4^	6.7 × 10^4^ ± 2.2 × 10^3^	4.9 × 10^2^ ± 20	**2.3** × **10**^**4**^ ± **3.8** × **10**^**2**^ *	14 ± 1.0	4.5 × 10^3^ ± 1.7 × 10^2^	1.5 × 10^4^ ± 8.5 × 10^2^	**33** ± **1.9** *
Olfactory Bulb	0 mg Cr(VI)/L (*n* = 7)	1.8 × 10^6^ ± 3.8 × 10^4^	1.6 × 10^5^ ± 4.2 × 10^3^	3.1 × 10^6^ ± 2.4 × 10^4^	3.2 × 10^5^ ± 1.9 × 10^5^	3.8 × 10^2^ ± 23	2.7 × 10^4^ ± 9.7 × 10^2^	15 ± 0.74	3.4 × 10^3^ ± 3.6 × 10^2^	1.6 × 10^4^ ± 6.4 × 10^2^	**28** ± **0.81** *
	5 mg Cr(VI)/L (*n* = 10)	1.8 × 10^6^ ± 4.5 × 10^4^	1.7 × 10^5^ ± 9.8 × 10^3^	3.0 × 10^6^ ± 7.4 × 10^4^	1.0 × 10^6^ ± 4.8 × 10^5^	3.6 × 10^2^ ± 15	2.6 × 10^4^ ± 1.1 × 10^3^	14 ± 0.49	3.2 × 10^3^ ± 1.1 × 10^2^	1.7 × 10^4^ ± 8.1 × 10^2^	**26** ± **1.2** *
Pituitary Gland	0 mg Cr(VI)/L (*n* = 9)	1.8 × 10^6^ ± 7.8 × 10^4^	2.3 × 10^5^ ± 1.7 × 10^4^	3.1 × 10^6^ ± 7.6 × 10^4^	1.0 × 10^6^ ± 5.5 × 10^5^	6.8 × 10^2^ ± 52	**6.5** × **10**^**4**^ ± **2.7** × **10**^**3**^ *	18 ± 1.3	**3.2** × **10**^**3**^ ± **1.0** × **10**^**2**^ *	2.3 × 10^4^ ± 9.8 × 10^2^	43 ± 1.9
	5 mg Cr(VI)/L (*n* = 10)	1.8 × 10^6^ ± 8.5 × 10^4^	2.0 × 10^5^ ± 1.1 × 10^4^	3.0 × 10^6^ ± 1.5 × 10^5^	3.7 × 10^5^ ± 9.6 × 10^4^	6.2 × 10^2^ ± 46	**5.8** × **10**^**4**^ ± **2.6** × **10**^**3**^ *	19 ± 1.9	**2.8** × **10**^**3**^ ± **1.5** × **10**^**2**^ *	2.3 × 10^5^ ± 1.4 × 10^3^	38 ± 2.8
Striatum	0 mg Cr(VI)/L (*n* = 9)	1.2 × 10^6^ ± 2.4 × 10^4^	1.8 × 10^5^ ± 3.3 × 10^3^	3.7 × 10^6^ ± 8.4 × 10^4^	6.1 × 10^4^ ± 1.9 × 10^3^	3.2 × 10^2^ ± 10	2.8 × 10^4^ ± 9.1 × 10^2^	**17** ± **0.61** *	3.1 × 10^3^ ± 1.6 × 10^2^	1.7 × 10^4^ ± 6.2 × 10^2^	**32** ± **1.3** *
	5 mg Cr(VI)/L (*n* = 10)	1.3 × 10^6^ ± 2.0 × 10^4^	1.8 × 10^5^ ± 2.5 × 10^3^	3.6 × 10^6^ ± 6.3 × 10^4^	6.8 × 10^4^ ± 3.9 × 10^3^	3.2 × 10^2^ ± 9.9	3.0 × 10^4^ ± 3.5 × 10^3^	**15** ± **0.67** *	3.2 × 10^3^ ± 92	1.6 × 10^4^ ± 7.6 × 10^2^	**29** ± **1.6** *

Data are reported as mean (ng/g) ± standard error of the mean. Bold font and (*) denote *p* < 0.1 between control guinea pigs and Cr(VI)-exposed guinea pigs.

**Table 4.** Essential metal levels in the brain by sex.

Sex	Region	Exposure Group	Sodium	Magnesium	Potassium	Calcium	Manganese	Iron	Cobalt	Copper	Zinc	Molybdenum
Males	Amygdala	0 mg Cr(VI)/L (*n* = 5)	1.5 × 10^6^ ± 1.3 × 10^5^	1.9 × 10^5^ ± 1.5 × 10^4^	4.2 × 10^6^ ± 2.7 × 10^5^	1.2 × 10^5^ ± 1.5 × 10^4^	4.3 × 10^2^ ± 47	3.4 × 10^4^ ± 1.2 × 10^4^	23 ± 3.0^a^	5.2 × 10^3^ ± 6.9 × 10^2^	3.3 × 10^4^ ± 3.4 × 10^3^	36 ± 2.5^a,c^
		5 mg Cr(VI)/L (*n* = 5)	1.3 × 10^6^ ± 2.9 × 10^4^	1.7 × 10^5^ ± 4.4 × 10^3^	3.8 × 10^6^ ± 9.6 × 10^4^	1.0 × 10^5^ ± 9.8 × 10^3^	3.5 × 10^2^ ± 18	1.9 × 10^4^ ± 1.0 × 10^3^	15 ± 1.3^a^	4.2 × 10^3^ ± 4.0 × 10^2^	2.7 × 10^4^ ± 8.6 × 10^2^	26 ± 1.4^a^
	Brain Stem	0 mg Cr(VI)/L (*n* = 5)	7.0 × 10^5^ ± 9.7 × 10^3^	8.6 × 10^4^ ± 2.6 × 10^3^	1.6 × 10^6^ ± 2.4 × 10^4^	8.4 × 10^4^ ± 5.2 × 10^4^	2.1 × 10^2^ ± 4.1	1.3 × 10^4^ ± 9.8 × 10^2^	4.8 ± 0.12^a^	1.6 × 10^3^ ± 1.2 × 10^2^	5.0 × 10^3^ ± 1.4 × 10^2^	14 ± 0.81^a^
		5 mg Cr(VI)/L (*n* = 5)	6.9 × 10^5^ ± 7.3 × 10^3^	8.3 × 10^4^ ± 6.3 × 10^2 d^	1.6 × 10^6^ ± 2.2 × 10^4^	6.7 × 10^4^ ± 1.8 × 10^4^	2.0 × 10^2^ ± 6.8	1.3 × 10^4^ ± 3.3 × 10^2^	4.1 ± 0.15^a^	1.5 × 10^3^ ± 81	4.9 × 10^3^ ± 51	12 ± 0.70^a^
	Cerebellum	0 mg Cr(VI)/L (*n* = 5)	7.2 × 10^5^ ± 2.2 × 10^4^	8.9 × 10^4^ ± 3.5 × 10^3^	1.7 × 10^6^ ± 1.1 × 10^4^	2.7 × 10^5^ ± 1.5 × 10^5^	2.7 × 10^2^ ± 6.9	1.2 × 10^4^ ± 6.5 × 10^2^	5.7 ± 0.11^a^	1.7 × 10^3^ ± 1.0 × 10^2 c^	6.2 × 10^3^ ± 1.1 × 10^2^	13 ± 0.40
		5 mg Cr(VI)/L (*n* = 5)	7.4 × 10^5^ ± 1.3 × 10^4^	1.0 × 10^5^ ± 7.8 × 10^3 d^	1.7 × 10^6^ ± 2.5 × 10^4^	8.3 × 10^5^ ± 4.3 × 10^5^	2.7 × 10^2^ ± 8.9	1.3 × 10^4^ ± 8.8 × 10^2 d^	5.1 ± 0.053^a^	1.6 × 10^3^ ± 74^d^	6.7 × 10^3^ ± 2.8 × 10^2 d^	12 ± 0.87
	Entorhinal Cortex	0 mg Cr(VI)/L (*n* = 5)	7.8 × 10^5^ ±1.0 × 10^4^	8.3 × 10^4^ ± 1.7 × 10^3^	1.8 × 10^6^ ± 3.3 × 10^4^	3.9 × 10^4^ ± 9.2 × 10^3^	1.6 × 10^2^ ± 2.8	9.3 × 10^3^ ± 5.5 × 10^2^	5.9 ± 0.19^a,c^	1.4 × 10^3^ ± 63	1.0 × 10^4^ ± 3.0 × 10^2^	13 ± 0.61^a^
		5 mg Cr(VI)/L (*n* = 5)	7.6 × 10^5^ ± 2.3 × 10^4^	8.5 × 10^4^ ± 7.4 × 10^2^	1.8 × 10^6^ ± 2.3 × 10^4^	5.8 × 10^4^ ± 2.2 × 10^4^	1.5 × 10^2^ ± 3.5	9.4 × 10^3^ ± 6.1 × 10^2^	4.8 ± 0.044^a^	1.4 × 10^3^ ± 81	9.3 × 10^3^ ± 3.4 × 10^2^	10 ± 0.63^a^
	Frontal Cortex	0 mg Cr(VI)/L (*n* = 5)	6.9 × 10^5^ ± 1.7 × 10^4^	8.6 × 10^4^ ± 1.1 × 10^3^	1.8 × 10^6^ ± 2.9 × 10^4^	1.3 × 10^5^ ± 2.7 × 10^4 c^	1.6 × 10^2^ ± 5.7^c^	9.8 × 10^3^ ± 3.7 × 10^2^	5.9 ± 0.21^a,c^	1.3 × 10^3^ ± 76	7.9 × 10^3^ ± 2.0 × 10^2^	13 ± 0.55^c^
		5 mg Cr(VI)/L (*n* = 5)	6.9 × 10^5^ ± 2.2 × 10^4^	9.4 × 10^4^ ± 7.5 × 10^3^	1.8 × 10^6^ ± 1.3 × 10^4^	6.3 × 10^5^ ± 4.5 × 10^5 d^	1.6 × 10^2^ ± 6.4	9.1 × 10^3^ ± 4.4 × 10^2^	5.0 ± 0.098^a^	1.2 × 10^3^ ± 96	8.0 × 10^3^ ± 3.6 × 10^2^	12 ± 0.67
	Dorsal Hippocampus	0 mg Cr(VI)/L (*n* = 5)	1.2 × 10^6^ ± 3.0 × 10^4^	1.5 × 10^5^ ± 1.3 × 10^3^	3.2 × 10^6^ ± 4.0 × 10^4^	5.8 × 10^4^ ± 1.7 × 10^3^	3.3 × 10^2^ ± 4.5	1.8 × 10^4^ ± 3.0 × 10^3^	9.7 ± 0.43	3.0 × 10^3^ ± 1.9 × 10^2^	1.9 × 10^4^ ± 5.4 × 10^2 c^	23 ± 1.3
		5 mg Cr(VI)/L (*n* = 5)	1.2 × 10^6^ ± 4.6 × 10^4^	1.5 × 10^5^ ± 2.5 × 10^3 d^	3.2 × 10^6^ ± 7.0 × 10^4^	6.1 × 10^4^ ± 3.6 × 10^3^	3.4 × 10^2^ ± 20	1.7 × 10^4^ ± 1.7 × 10^3^	9.2 ± 0.61	3.1 × 10^3^ ± 1.4 × 10^2^	1.7 × 10^4^ ± 1.8 × 10^3^	22 ± 0.97
	Ventral Hippocampus	0 mg Cr(VI)/L (*n* = 5)	1.2 × 10^6^ ± 3.9 × 10^4^	1.4 × 10^5^ ± 7.5 × 10^3^	3.0 × 10^6^ ± 1.9 × 10^5^	9.0 × 10^4^ ± 3.1 × 10^4^	3.4 × 10^2^ ± 24	1.8 × 10^4^ ± 1.6 × 10^3^	10 ± 0.78	3.1 × 10^3^ ± 1.8 × 10^2^	1.7 × 10^4^ ± 1.5 × 10^3^	21 ± 1.7
		5 mg Cr(VI)/L (*n* = 5)	1.2 × 10^6^ ± 2.5 × 10^4^	1.4 × 10^5^ ± 1.5 × 10^3^	3.0 × 10^6^ ± 2.8 × 10^4^	6.0 × 10^4^ ± 4.1 × 10^3^	3.3 × 10^2^ ± 7.8	2.1 × 10^4^ ± 2.8 × 10^3^	9.3 ± 0.39	3.2 × 10^3^ ± 1.2 × 10^2^	1.8 × 10^4^ ± 1.5 × 10^3^	19 ± 1.5
	Hypothalamus	0 mg Cr(VI)/L (*n* = 5)	1.4 × 10^6^ ± 8.9 × 10^4^	1.6 × 10^5^ ± 9.7 × 10^3^	3.6 × 10^6^ ± 1.9 × 10^5^	1.9 × 10^5^ ± 1.2 × 10^5^	5.2 × 10^2^ ± 34	2.5 × 10^4^ ± 1.3 × 10^3^	17 ± 2.3	4.3 × 10^3^ ± 1.7 × 10^2^	1.9 × 10^4^ ± 2.8 × 10^3^	40 ± 2.5^a^
		5 mg Cr(VI)/L (*n* = 5)	1.3 × 10^6^ ± 3.4 × 10^4^	1.6 × 10^5^ ± 2.0 × 10^3^	3.5 × 10^6^ ± 4.4 × 10^4 d^	6.5 × 10^4^ ± 2.5 × 10^3^	4.9 × 10^2^ ± 29	2.3 × 10^4^ ± 5.3 × 10^2^	14 ± 1.9	4.6 × 10^3^ ± 2.8 × 10^2^	1.5 × 10^4^ ± 6.6 × 10^2^	31 ± 1.9^a^
	Olfactory Bulb	0 mg Cr(VI)/L (*n* = 4)	1.8 × 10^6^ ± 7.0 × 10^4^	1.7 × 10^5^ ± 7.1 × 10^3^	3.1 × 10^6^ ± 3.0 × 10^4 c^	4.7 × 10^5^ ± 3.3 × 10^5^	4.2 × 10^2^ ± 30	2.7 × 10^4^ ± 1.7 × 10^3^	16 ± 0.74	3.6 × 10^3^ ± 6.4 × 10^2^	1.5 × 10^4^ ± 6.3 × 10^2^	30 ± 0.85^a,c^
		5 mg Cr(VI)/L (*n* = 5)	1.9 × 10^6^ ± 7.1 × 10^4^	1.7 × 10^5^ ± 4.6 × 10^3^	3.1 × 10^6^ ± 3.8 × 10^4 d^	4.9 × 10^5^ ± 2.4 × 10^5^	3.6 × 10^2^ ± 18	2.7 × 10^4^ ± 1.4 × 10^3^	14 ± 0.77	3.4 × 10^3^ ± 1.6 × 10^2 d^	1.8 × 10^4^ ± 1.2 × 10^3^	26 ± 1.5^a^
	Pituitary Gland	5 mg Cr(VI)/L (*n* = 5)	1.9 × 10^6^ ± 8.4 × 10^4^	2.5 × 10^5^ ± 2.4 × 10^4 c^	3.2 × 10^6^ ± 1.0 × 10^5^	1.7 × 10^6^ ± 9.4 × 10^5 c^	7.9 × 10^2^ ± 48^c^	6.5 × 10^4^ ± 4.1 × 10^3^	21 ± 1.7^c^	3.3 × 10^3^ ± 1.1 × 10^2^	2.5 × 10^4^ ± 8.0 × 10^2^	47 ± 1.1^c^
		0 mg Cr(VI)/L (*n* = 5)	1.9 × 10^6^ ± 3.9 × 10^4^	2.2 × 10^5^ ± 6.5 × 10^3^	3.1 × 10^6^ ± 5.6 × 10^4^	5.3 × 10^5^ ± 1.7 × 10^5^	6.9 × 10^2^ ± 47	5.7 × 10^4^ ± 3.3 × 10^3^	18 ± 1.1	3.1 × 10^3^ ± 1.1 × 10^2^	2.3 × 10^4^ ± 1.2 × 10^3^	43 ± 2.7^d^
	Striatum	5 mg Cr(VI)/L (*n* = 5)	1.3 × 10^6^ ± 2.9 × 10^4^	1.8 × 10^5^ ± 5.6 × 10^3^	3.7 × 10^6^ ± 1.4 × 10^5^	6.0 × 10^4^ ± 1.7 × 10^3^	3.3 × 10^2^ ± 14	2.8 × 10^4^ ± 1.4 × 10^3^	17 ± 0.66	3.1 × 10^3^ ± 2.6 × 10^2^	1.7 × 10^4^ ± 7.3 × 10^2^	33 ± 2.0^a^
		0 mg Cr(VI)/L (*n* = 5)	1.3 × 10^6^ ± 1.5 × 10^4^	1.8 × 10^5^ ± 2.1 × 10^3^	3.7 × 10^6^ ± 6.4 × 10^4^	6.9 × 10^4^ ± 7.6 × 10^3^	3.2 × 10^2^ ± 15	3.4 × 10^4^ ± 6.7 × 10^3^	15 ± 0.84	3.2 × 10^3^ ± 1.3 × 10^2^	1.6 × 10^4^ ± 9.0 × 10^2^	28 ± 0.68^a^

Data are reported as mean (ng/g) ± standard error of the mean.

(a) *p* < 0.1 between control males and Cr(VI)-exposed males;

(c) *p* < 0.1 between control males and control females;

(d) *p* < 0.1 between Cr(VI)-exposed males and Cr(VI)-exposed females.

## Data Availability

The original contributions presented in this study are included in the article/Supplementary Material. Further inquiries can be directed to the corresponding author.
