# Supplementary material for: Subchronic Exposure to Hexavalent Chromium in Drinking-Water-Induced Sex-Dependent Essential Metal Dyshomeostasis in the Liver, Blood, and Brain of Guinea Pigs
Source: Livers. Author manuscript; Available in PMC 2026 Nov 11. (PMC13750741; doi:10.3390/livers6050090)
Supplement: Supplementary file 1 [file NIHMS2219048-supplement-Supp_Material.pdf]

Table S1. Quality assurance and quality control analysis of liver and blood samples analyzed on an Agilent 7900 ICP-MS by the Integrative Molecular Analysis Core at the University of New Mexico.

Essential Metal	Method Detection Limit (ppb)	Calibration Standard 1 (ppb)	Calibration Standard 2 (ppb)	Calibration Standard 3 (ppb)	Calibration Standard 4 (ppb)	Calibration Standard 5 (ppb)	Calibration Standard 6 (ppb)	Calibration Curve R^2-Value	Continuing Calibration Verification (% Recovery ± SEM)	NIST 1643f Reference Material (% Recovery)
Chromium	0.013292	0	0.13	1.09	9.93	101.60	999.84	1.00	103.58 ± 0.43	108.75
Sodium	158.7341	0	13.99	95.25	1141.72	11080.58	99890.53	1.00	80.77 ± 3.14	96.94
Magnesium	15.12249	0	10.55	103.12	934.12	11019.96	99898.66	1.00	112.44 ± 0.39	97.24
Potassium	198.3054	0	10.77	107.94	1024.46	11132.14	99886.53	1.00	87.23 ± 1.25	104.11
Calcium	10.01811	0	30.26	266.14	1177.67	10428.51	99955.20	1.00	93.25 ± 0.39	103.01
Manganese	0.23428	0	0.59	1.19	10.21	104.52	999.55	1.00	101.45 ± 0.40	105.54
Iron	15.48901	0	12.12	108.89	1084.50	10826.04	99916.54	1.00	95.71 ± 0.58	99.01
Cobalt	0.022934	0	0.11	1.08	10.40	103.57	999.64	1.00	102.35 ± 0.61	104.62
Copper	2.692075	0	0.24	1.63	11.11	108.01	999.19	1.00	89.76 ± 0.65	101.15
Zinc	2.685761	0	1.09	2.94	12.71	118.30	998.14	0.99	75.21 ± 0.69	93.69
Molybdenum	0.184322	0	0.09	0.96	8.73	97.57	1000.26	1.00	124.16 ± 0.84	110.87

Table S2. Quality assurance and quality control analysis of brain samples analyzed on an Agilent 7900 ICP-MS by the Metallomics Core at the University of Louisville

Essential Metal	Method Detection Limit (ppb)	Calibration Standard 1 (ppb)	Calibration Standard 2 (ppb)	Calibration Standard 3 (ppb)	Calibration Standard 4 (ppb)	Calibration Standard 5 (ppb)	Calibration Standard 6 (ppb)	Calibration Curve R^2-Value	Continuing Calibration Verification (% Recovery ± SEM)
Chromium	0.03861	0	1.08	2.47	5.08	10.52	49.87	1.00	89.61 ± 0.86
Sodium	5.056	0	97.65	199.99	493.12	1026.58	4995.42	1.00	89.79 ± 1.04
Magnesium	0.2658	0	113.37	229.12	551.04	1130.19	5341.39	0.98	81.18 ± 0.99
Potassium	8.852	0	96.98	206.09	504.43	1023.49	4994.68	1.00	90.15 ± 0.77
Calcium	3.72	0	114.67	220.16	519.84	1023.99	4992.12	1.00	88.82 ± 0.97
Manganese	0.01259	0	1.06	2.17	5.04	10.53	49.88	1.00	89.76 ± 0.81
Iron	0.03389	0	105.79	213.09	524.82	1079.98	4980.83	1.00	86.92 ± 1.06
Cobalt	0.0006494	0	1.07	2.14	5.18	10.53	49.87	1.00	88.60 ± 0.87
Copper	0.008171	0	1.36	2.25	5.16	10.56	49.85	1.00	82.13 ± 0.83
Zinc	0.03123	0	1.35	2.32	5.14	10.53	49.86	1.00	90.12 ± 0.91
Molybdenum	0.0000388	0	1.00	2.09	4.99	10.23	49.95	1.00	93.52 ± 0.66

Table S3. Pooled essential metal levels in the liver.

Exposure Group	Sodium	Magnesium	Potassium	Calcium	Manganese	Iron	Cobalt	Copper	Zinc	Molybdenum
0 mg Cr(VI)/L (*n* = 17)	$9.0\times10^{5} \pm 4.8\times10^{4}$	**$1.8\times10^{5} \pm 3.5\times10^{3}$***	**$2.5\times10^{6} \pm 8.4\times10^{4}$***	$3.6\times10^{4} \pm 2.2\times10^{3}$	$2.5\times10^{3} \pm 1.3\times10^{2}$	$1.7\times10^{5} \pm 1.4\times10^{4}$	55 ± 2.1	$1.6\times10^{4} \pm 1.6\times10^{3}$	**$2.8\times10^{4} \pm 8.8\times10^{2}$***	**$7.4\times10^{2} \pm 28$***
5 mg Cr(VI)/L (*n* = 20)	$1.1\times10^{6} \pm 6.1\times10^{4}$	**$1.7\times10^{5} \pm 3.5\times10^{3}$***	**$2.2\times10^{6} \pm 9.2\times10^{4}$***	$3.7\times10^{4} \pm 2.2\times10^{3}$	$2.3\times10^{3} \pm 71$	$1.6\times10^{5} \pm 1.0\times10^{4}$	52 ± 1.6	$1.7\times10^{4} \pm 1.7\times10^{3}$	**$2.6\times10^{4} \pm 6.0\times10^{2}$***	**$6.8\times10^{3} \pm 17$***

Data are reported as mean (ng/g) ± standard error of the mean. Bold text and (*) denote $p < 0.1$ between control guinea pigs and Cr(VI)-exposed guinea pigs.

Table S4. Sex-matched essential metal levels in the liver.

Sex	Exposure Group	Sodium	Magnesium	Potassium	Calcium	Manganese	Iron	Cobalt	Copper	Zinc	Molybdenum
Male	0 mg Cr(VI)/L (*n* = 9)	$9.0\times10^{5} \pm 5.2\times10^{4}$	$1.7\times10^{5} \pm 4.9\times10^{3}$ [c]	$2.5\times10^{6} \pm 9.7\times10^{4}$	$3.0\times10^{4} \pm 1.3\times10^{3}$ [c]	$2.4\times10^{3} \pm 1.7\times10^{2}$	$1.3\times10^{5} \pm 5.2\times10^{3}$ [c]	52 ± 2.4 [c]	$1.5\times10^{4} \pm 2.4\times10^{3}$	$2.7\times10^{4} \pm 1.2\times10^{3}$	$7.7\times10^{2} \pm 44$
	5 mg Cr(VI)/L (*n* = 10)	$1.1\times10^{6} \pm 9.5\times10^{4}$	$1.6\times10^{5} \pm 4.4\times10^{3}$	$2.2\times10^{6} \pm 1.4\times10^{5}$	$3.4\times10^{4} \pm 2.9\times10^{3}$	$2.3\times10^{3} \pm 1.0\times10^{2}$	$1.5\times10^{5} \pm 1.2\times10^{4}$	47 ± 1.7 [d]	$1.4\times10^{4} \pm 2.4\times10^{3}$ [d]	$2.5\times10^{4} \pm 6.0\times10^{2}$	$7.0\times10^{2} \pm 28$
Female	0 mg Cr(VI)/L (*n* = 8)	$9.0\times10^{5} \pm 8.7\times10^{4}$	$1.8\times10^{5} \pm 4.3\times10^{3}$ [b,c]	$2.6\times10^{6} \pm 1.5\times10^{5}$ [b]	$4.2\times10^{4} \pm 3.5\times10^{3}$ [c]	$2.5\times10^{3} \pm 2.0\times10^{2}$	$2.1\times10^{5} \pm 1.9\times10^{4}$ [c]	59 ± 2.9 [c]	$1.7\times10^{4} \pm 2.2\times10^{3}$	$2.9\times10^{4} \pm 1.3\times10^{3}$ [b]	$7.0\times10^{2} \pm 30$
	5 mg Cr(VI)/L (*n* = 10)	$1.1\times10^{6} \pm 8.2\times10^{4}$	$1.7\times10^{5} \pm 5.6\times10^{3}$ [b]	$2.3\times10^{6} \pm 1.2\times10^{5}$ [b]	$4.0\times10^{4} \pm 3.1\times10^{3}$	$2.3\times10^{3} \pm 1.0\times10^{2}$	$1.7\times10^{5} \pm 1.6\times10^{4}$	57 ± 1.7 [d]	$1.9\times10^{4} \pm 2.2\times10^{3}$ [d]	$2.6\times10^{4} \pm 1.1\times10^{3}$ [b]	$6.5\times10^{2} \pm 17$

Data are reported as mean (ng/g) ± standard error of the mean. [(a)] $p < 0.1$ between control males Cr(VI)-exposed males; [(b)] $p < 0.1$ between control females and Cr(VI)-exposed females; [(c)] $p < 0.1$ between control males and control females; [(d)] $p < 0.1$ between Cr(VI)-exposed males and Cr(VI)-exposed females.

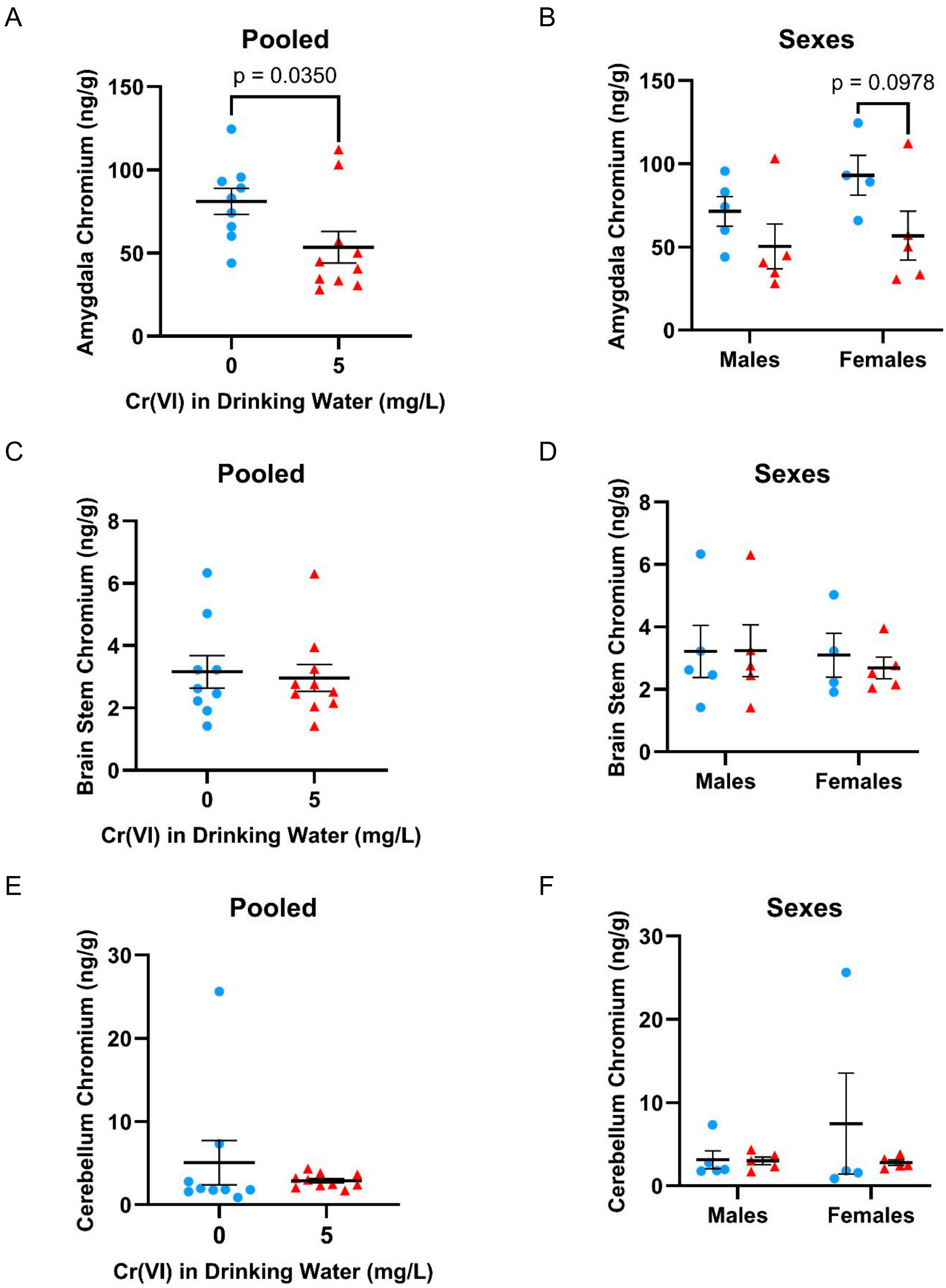

A
Pooled
p = 0.0350
Amygdala Chromium (ng/g)
150
100
50
0
0
5
Cr(VI) in Drinking Water (mg/L)
B
Sexes
p = 0.0978
Amygdala Chromium (ng/g)
150
100
50
0
Males
Females
C
Pooled
Brain Stem Chromium (ng/g)
8
6
4
2
0
0
5
Cr(VI) in Drinking Water (mg/L)
D
Sexes
Brain Stem Chromium (ng/g)
8
6
4
2
0
Males
Females
E
Pooled
Cerebellum Chromium (ng/g)
30
20
10
0
0
5
Cr(VI) in Drinking Water (mg/L)
F
Sexes
Cerebellum Chromium (ng/g)
30
20
10
0
Males
Females

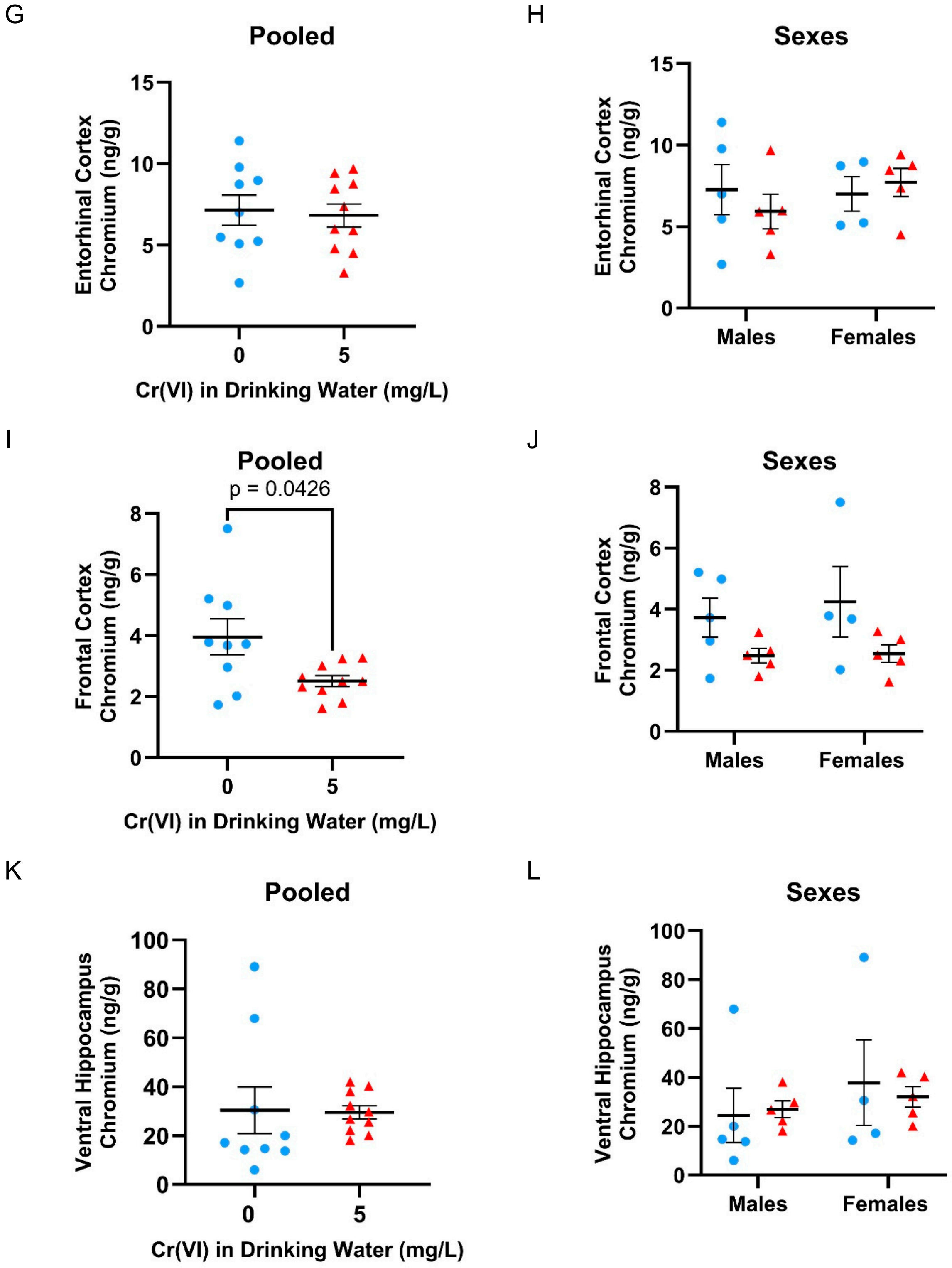
G
Pooled
Entorhinal Cortex Chromium (ng/g)
15
10
5
0
0
5
Cr(VI) in Drinking Water (mg/L)
H
Sexes
Entorhinal Cortex Chromium (ng/g)
15
10
5
0
Males
Females
I
Pooled
p = 0.0426
Frontal Cortex Chromium (ng/g)
8
6
4
2
0
0
5
Cr(VI) in Drinking Water (mg/L)
J
Sexes
Frontal Cortex Chromium (ng/g)
8
6
4
2
0
Males
Females
K
Pooled
Ventral Hippocampus Chromium (ng/g)
100
80
60
40
20
0
0
5
Cr(VI) in Drinking Water (mg/L)
L
Sexes
Ventral Hippocampus Chromium (ng/g)
100
80
60
40
20
0
Males
Females

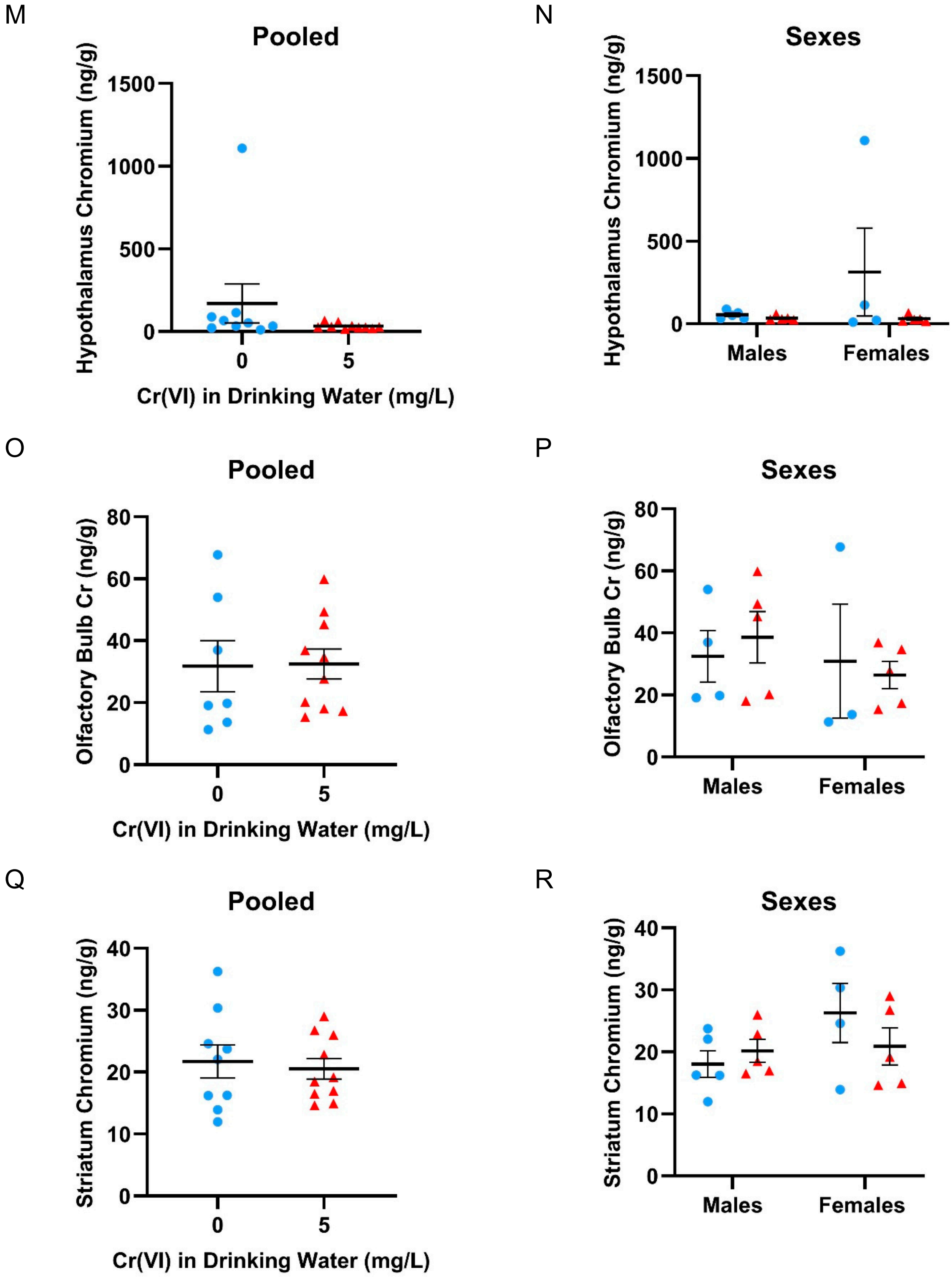

Figure S1. Chromium did not significantly accumulate in other isolated brain regions. Total chromium in the **A)** amygdala; **B)** amygdala, separated by sex; **C)** brain stem; **D)** brainstem, separated by sex; **E)** cerebellum; **F)** cerebellum, separated by sex; **G)** entorhinal cortex; **H)** entorhinal cortex, separated by sex; **I)** frontal cortex; **J)** frontal cortex, separated by sex; **K)** ventral hippocampus; **L)** ventral hippocampus, separated by sex; **M)** hypothalamus; **N)** hypothalamus, separated by sex; **O)** olfactory bulb; **P)** olfactory bulb, separated by sex; **Q)** striatum; **R)** striatum, separated by sex. Each dot represents one animal, with blue circles representing animals in the control group and red triangles representing animals exposed to 5 mg Cr(VI)/L in drinking water. Data are reported as mean ± standard error of the mean (*n* = 7-10 guinea pigs per exposure group and 3-5 per sex.)
